# The global gap in treatment coverage for major depressive disorder in 84 countries from 2000–2019: A systematic review and Bayesian meta-regression analysis

**DOI:** 10.1371/journal.pmed.1003901

**Published:** 2022-02-15

**Authors:** Modhurima Moitra, Damian Santomauro, Pamela Y. Collins, Theo Vos, Harvey Whiteford, Shekhar Saxena, Alize J. Ferrari

**Affiliations:** 1 Institute for Health Metrics and Evaluation, University of Washington, Seattle, Washington, United States of America; 2 Department of Global Health, University of Washington, Seattle, Washington, United States of America; 3 The University of Queensland, School of Public Health, Brisbane, Queensland, Australia; 4 Queensland Centre for Mental Health Research, Brisbane, Queensland, Australia; 5 Department of Psychiatry and Behavioral Sciences, University of Washington, Seattle, Washington, United States of America; 6 Harvard T H Chan School of Public Health, Harvard University, Boston, Massachusetts, United States of America; Addis Ababa University / King’s College London, ETHIOPIA

## Abstract

**Background:**

The treatment coverage for major depressive disorder (MDD) is low in many parts of the world despite MDD being a major contributor to disability globally. Most existing reviews of MDD treatment coverage do not account for potential sources of study-level heterogeneity that contribute to variation in reported treatment rates. This study aims to provide a comprehensive review of the evidence and analytically quantify sources of heterogeneity to report updated estimates of MDD treatment coverage and gaps by location and treatment type between 2000 and 2019.

**Methods and findings:**

A systematic review of the literature was conducted to identify relevant studies that provided data on treatment rates for MDD between January 1, 2000, and November 26, 2021, from 2 online scholarly databases PubMed and Embase. Cohort and cross-sectional studies were included if treatment rates pertaining to the last 12 months or less were reported directly or if sufficient information was available to calculate this along with 95% uncertainty intervals (UIs). Studies were included if they made use of population-based surveys that were representative of communities, countries, or regions under study. Studies were included if they used established diagnostic criteria to diagnose cases of MDD. Sample and methodological characteristics were extracted from selected studies. Treatment rates were modeled using a Bayesian meta-regression approach and adjusted for select covariates that quantified heterogeneity in the data. These covariates included age, sex, treatment type, location, and choice of MDD assessment tool. A total of 149 studies were included for quantitative analysis. Treatment coverage for health service use ranged from 51% [95% UI 20%, 82%] in high-income locations to 20% [95% UI 1%, 53%] in low- and lower middle-income locations. Treatment coverage for mental health service use ranged from 33% [95% UI 8%, 66%] in high-income locations to 8% [95% UI <1%, 36%] in low- and lower middle-income countries. Minimally adequate treatment (MAT) rates ranged from 23% [95% UI 2%, 55%] in high-income countries to 3% [95% UI <1%, 25%]) in low- and lower middle-income countries. A primary methodological limitation was the lack of sufficient data from low- and lower middle-income countries, which precluded our ability to provide more detailed treatment rate estimates.

**Conclusions:**

In this study, we observed that the treatment coverage for MDD continues to be low in many parts of the world and in particular in low- and lower middle-income countries. There is a continued need for routine data collection that will help obtain more accurate estimates of treatment coverage globally.

## Abstract 2

In a systematic review and Bayesian meta-regression analysis, Modhurima Moitra and colleagues estimate major depressive disorder treatment coverage in 84 countries.

## Author summary

**Why was this study done?:**

➢ Major depressive disorder (MDD) is one of the major contributors to disability worldwide, but treatment rates for this condition are remarkably low.
➢ To the best of our knowledge, previous systematic reviews on this topic provide more descriptive summaries of treatment rates without accounting for differences in study attributes that may contribute to variation in reported treatment rates.
➢ An updated systematic review that is more reflective of the recent literature on treatment rates as well as an improved analytical approach may provide more accurate estimates of treatment rates by resource setting and geography.

**What did the researchers do and find?:**

➢ We conducted a systematic review and meta-regression analysis using data on treatment rates for MDD from 149 studies and 84 countries between 2000 and 2021.
➢ We estimated pooled treatment rates adjusted for parameters of interest including age, sex, treatment type, study methods, and location.
➢ Mental health service use ranged from 33% (95% uncertainty interval (UI) 8, 66) in high-income countries to 8% (95% UI <1, 36) in low- and lower middle-income countries.
➢ Minimally adequate treatment (MAT) ranged from 23% (95% UI 2, 55) in high-income countries to 3% (95% UI <1, 25) in low- and lower middle-income countries.

**What do these findings mean?:**

➢ This systematic review provides updated evidence on treatment rates for MDD, and results suggest that there are wide disparities in treatment rates by resource setting.
➢ More high-quality data on depression treatment coverage and adequacy are needed from low- and lower middle-income countries.
➢ These findings may help prioritize efforts to scale up depression treatment in locations with clearly identified treatment gaps.

## Introduction

Depressive disorders are highly prevalent and disabling. The Global Burden of Disease Study 2019 (GBD 2019) estimated that major depressive disorder (MDD) and dysthymia were jointly responsible for 46.9 million disability-adjusted life years (DALYs) globally in 2019, with each DALY equivalent to a healthy year of life lost to the disability caused by depressive disorders [1]. When benchmarked against a total of 369 diseases and injuries, depressive disorders were the 13th leading cause of overall burden and the seventh leading cause of nonfatal burden, globally [1,2]. The impact of depressive disorders also goes beyond the disability and mortality captured by the DALY. People with depressive disorders, caregivers, employers, and governments must manage the associated reductions in work productivity and increased reliance on state health and welfare services [3]. Depressive disorders are also known to be major risk factors for fatal outcomes such as suicide [4,5].

Effective and efficient treatment strategies are available for depressive disorders and consist of pharmacotherapy, psychological, and social interventions [6]. In recent years, there have been signs of increasing global commitment to prioritize mental health and reduce the burden imposed by severe forms of mental disorders such as MDD. In 2013, the World Health Assembly adopted the Comprehensive Mental Health Action Plan, which was extended to 2030 at the 72nd World Health Assembly [7,8]. Among the global targets set are for member states to increase service coverage for severe mental disorders by 20% by the year 2030 and to routinely collect information on key mental health indicators such as disorder prevalence and treatment. In 2015, the United Nations General Assembly passed the 2030 Agenda for Sustainable Development, which for the first time identified the promotion of mental health and well-being, and the prevention and treatment of substance abuse, as health priorities within the global development agenda [9].

These national and global advances suggest increasing commitment by governments to reduce the negative effects of mental and substance use disorders. However, despite depression being a major cause of disability, treatment rates for depression are remarkably low. An estimated 7% to 28% of those with depression receive appropriate care and treatment [3]. Previous reviews on depression treatment rates have found wide geographic variation by WHO region with gaps in treatment ranging from 45.4% in Europe to 67% in the African region and 70.2% in the Eastern Mediterranean region [10]. There also exists variation by resource setting in the quality of care received. The proportion of those receiving minimally adequate treatment (MAT, representing the combination of treatment strategies established by research to be minimally sufficient in treating those with depression) ranged from 22.4% in high-income countries to 3.7% in lower middle-income countries [11].

Health information systems in many countries are not designed to routinely collect data on key mental health indicators (such as treatment coverage) from which the extent of any progress can be measured [12]. As an alternative, we can turn to population-representative epidemiological surveys to estimate the treated and untreated prevalence of depressive disorders as an indicator of treatment gaps. While epidemiological surveys capturing data on service use for individuals with depressive disorders exist, efforts to assemble and critically evaluate the data for a representative global summary of treatment rates are outdated or do not capture all available information [13]. Furthermore, most existing reviews rely on a descriptive summarization of treatment rates or gaps, without accounting for variation in study methodology that may potentially contribute to heterogeneity in the existing evidence, thereby resulting in imprecise estimates.

In this paper, we sought to update the work of Kohn and colleagues who undertook a literature review of population surveys of mental and substance use disorders for data on the proportion of individuals receiving care [10]. Treatment gap here referred to the difference between the proportion of the individuals within a given population with a mental disorder (i.e., total prevalent cases) and the proportion of these individuals who received treatment for that disorder (i.e., treated prevalent cases). Having been conducted more than 16 years ago, more recent data on the prevalence and treatment rates of depressive disorders can be used to derive treatment gaps estimates that consider the following: (1) the increased availability of data for some regions; (2) recent health reforms that may have occurred in some countries that influence treatment rates; and (3) changes in the use of some interventions that could affect treatment rates. In this study, we update the knowledge base on the gaps in treatment coverage for MDD. We conducted a systematic literature review to identify the existing literature on the global treatment rates of MDD. Potential sources of heterogeneity were analytically explored and accounted for to generate predicted treatment rates. These were combined with population-representative prevalence estimates derived by GBD 2019 to estimate treatment gaps for MDD.

## Methods

### Case definitions

This study focused on MDD as defined by the Diagnostic and Statistical Manual of Mental Disorders (DSM versions III, IV, or 5) or the International Classification of Diseases (ICD versions 9 or 10) diagnostic criteria [14,15]. According to the DSM, MDD is an episodic disorder characterized by at least one major depressive episode (MDE) in the past 12 months. A MDE involves symptoms of depressed mood and/or loss of interest causing clinically significant impairment in the main areas of functioning. The equivalence as defined by the ICD-10 is characterized by at least 2 of the following symptoms: depressed mood, loss of interest, and/or fatigue (ICD-10: F-32). To meet the threshold for a diagnosis of MDD, depressed mood, anhedonia, or fatigue must be experienced mostly all day and every day for a minimum period of 2 weeks.

#### Treatment rates

Treatment rates were defined as the proportion of cases of MDD that received treatment for the disorder. Types of treatment were classified into categories used in previous studies by Thornicroft and colleagues (2017) and Wang and colleagues (2007) [11,16]. These classifications were used to ensure consistent cross-national comparisons of the multiple sectors from which people may receive treatment. The treatment type categories used for this analysis are listed in Table 1 below.

**Table 1:** Treatment types and corresponding definitions.

Treatment type	Definition
Any service use	Studies that reported treatment rates without differentiating between health and nonhealth sectors
Health service use	Services offered within the health sector
General health service use	Services provided by primary care doctors, other general medical doctors, nurses, or other health professionals not within the mental health sector
Mental health service use	Services provided by psychiatrists, psychologists, other mental health professionals in any setting, social workers, or counselors in a mental health specialty setting or use of a mental health hotline
Nonhealth service use	Services outside of the health sector. This includes service provided by spiritual or religious advisers, chiropractors, traditional healers, participation in internet support groups, and self-help groups
MAT	Treatment that was potentially minimally adequate according to evidence-based guidelines. Due to the variation in the definition of MAT between studies, we chose to group definitions as being “stringent” or “nonstringent”. Stringent MAT was defined as receiving 8 or more visits to any service sector for psychotherapy or 4 or more visits to any service sector and at least 30 days of pharmacotherapy or its nearest equivalent. Nonstringent MAT was defined as requiring fewer visits and days of medication use than the stringent definition of MAT. These groups best reflected the variation observed in the reported definitions of MAT

MAT, minimally adequate treatment.

### Data sources and search strategy

The estimation of treatment gaps required data on prevalence and treatment rates for MDD. Prevalence data came from work undertaken as part of the GBD 2019 study. A systematic literature review of the treatment rates data was undertaken as part of this present study.

#### Disorder-specific treatment rates

A new systematic review was conducted to capture information on treatment rates, using methods that would ensure that estimates were comparable with the GBD 2019 literature review and analysis of prevalence data. Searches were performed in 2 online scholarly databases Embase and PubMed from January 1, 2000, to November 26, 2021, including keywords such as “depres*” OR “dysthymia” AND “service OR care” AND “utilization” (see S1 Appendix, Section 1 for full search strings). No restrictions were placed on study language. An additional search of all data sources used to estimate the prevalence of MDD in GBD 2019 was conducted to ensure that all relevant data sources were screened (see more details on GBD data below). Both reviews adhered to the Preferred Reporting Items for Systematic Reviews and Meta-Analyses (PRISMA) guidelines [17] (see S1 Checklist and S1 Appendix, Section 2). For each of the 2 literature searches, potential data sources were assessed for inclusion through a title, abstract, and full text search, respectively. MM, AJF, JL, and KJ conducted the systematic review between 2000 and 2016. The review was updated to 2021 by MM, JS, PM, DS, and AJF (see Acknowledgments). All reviewers followed the same protocol for review and extraction. This included the use of the standardized data extraction sheet and inclusion and exclusion criteria. Studies assessed by different reviewers were cross-checked by the lead author. Discrepancies were discussed together with the senior authors in order for a consensus to be met. This systematic review is registered with PROSPERO (ID: CRD42020212552) [18].

GBD 2019 estimated the prevalence of MDD and dysthymia by age, sex, year, and location as part of their analysis of nonfatal burden. The GBD search strategy has been reported elsewhere and is summarized here [1]. Briefly, prevalence estimates were derived from an analysis of epidemiological population survey data obtained from comprehensive systematic reviews of the literature reporting on the prevalence, incidence, remission, duration, and excess mortality associated with MDD and dysthymia. The literature search involved examining the peer-reviewed literature (via PubMed, PsychInfo, and Embase) between 1980 and 2019 and obtaining other relevant data sources from the gray literature or through expert consultation up to 2019. To meet criteria for inclusion, studies reporting prevalence must have the following: defined a case of MDD or dysthymia using diagnostic classifications proposed in the DSM or ICD; involved/recruited a sample representative of the community, region, or country under study (i.e., samples of minority groups or those derived from hospital records were not accepted); and reported prevalence within the past year or less. Lifetime prevalence estimates were not accepted as they are more prone to recall bias [19].

The primary metric of interest for the review of treatment rates was the proportion of individuals from general population surveys, meeting criteria for MDD that received treatment for their disorder. Studies were included if (i) treatment rates pertaining to the last 12 months or less were reported directly or if sufficient information was available to calculate this along with 95% uncertainty intervals (UIs); (ii) they made use of population-based surveys that were representative of communities, countries, or regions under study; (iii) used DSM-III, DSM-IV, or DSM-5 or ICD-9 or ICD-10 criteria to diagnose cases of MDD; and (iv) reported data collected between 2000 and 2019 were included. Earlier samples were excluded, given that changes in the available treatment and service systems between countries have likely evolved over time. Therefore, treatment rates from years earlier than 2000 may not be representative of the current state of treatment coverage and service quality [20]. Studies were included if they met all inclusion criteria listed above. To maximize data availability, studies that reported on the current prevalence of “depressive disorders” (comprising both MDD and dysthymia) or “mood disorders” were included and reported on separately (see S1 Appendix, Section 7). Studies were excluded if they (i) exclusively reported on nonrepresentative samples (e.g., inpatient samples, perinatal women, incarcerated samples, populations without fixed residences, populations that are racial or ethnic minorities in the study location); or (ii) used symptom scales to assess for the presence of depressive symptoms not meeting diagnostic thresholds within the DSM and ICD. An exception to this criterion was made for data from the World Health Surveys (WHS), which were included and adjusted in order to maximize geographic representation of available data sources (see next section for more details).

In addition to our primary literature search, a gray literature search was also conducted as part of the review of GBD data to identify datasets from the WHS, which captured data on both depressive symptoms and service use across 70 countries. The survey items relating to depression in the WHS captured the majority but not all symptoms required for a full diagnosis of MDD according to the DSM or ICD. As such, the estimated treatment rates likely pertained to a combination of individuals with MDD and subthreshold MDD. Details on these data and adjustment of WHS estimates are reported in the appendix (See S1 Appendix, Section 3).

### Data collection and processing

Study quality and risk of bias were assessed as part of data extraction and analysis. Only studies meeting strict inclusion criteria were included in the analysis. Remaining sources of measurement error in estimates reported between studies were investigated as covariates within the regression analysis. Risk of publication bias was assessed using funnel plots (see S1 Appendix). Data from included studies were extracted using a Microsoft Excel template that ensured that minimum amount of information was extracted from each study. Study characteristics that were extracted included location, study setting, methodological design, urbanicity (mixed/rural/urban), years, and diagnostic tools used. Sample characteristics that were extracted included age, sex, response rate, treatment type, and sample size (see Table 2 for a full list of study characteristics extracted). Studies were classified by income level according to the World Bank Country and Lending Groups and GBD super regions [1,21]. If studies reported multiple treatment rates (e.g., stratified by age or sex), the estimate for each was extracted. Similarly, if studies reported multiple treatment rates by severity of MDD, these were extracted and analyzed separately. Estimates for treatment rates were stratified into categories of treatment types described above. Treatment rates are bounded by 0% and 100%, and some treatment rates and UIs may be close to these boundaries. Since normal approximations can result in impossible estimates near these boundaries (such as below 0% or above 100%), the Freeman–Tukey double arcsine transformation was used to stabilize variances in our dataset [22,23]. Pooled estimates were then back-transformed into natural number space and reported in the results section (see next section for more details).

**Table 2:** List of parameters extracted and definitions.

Parameter	Definition
Disorder	As reported by the study: MDD or dysthymia or depressive disorders or mood disorders (The main analyses focused on MDD only)
Country	As reported by study
World Bank Income Group	High-income (ref)/upper-middle/lower-middle/low-income
Year	Midpoint of duration between start and end years of study period
Age	Median age of sample reported by study
Percent female	Percentage of study sample that comprised female participants
Treatment type	Any service/health service/general health service/mental health service/nonhealth service
Survey instrument used to assess MDD	Mental disorder diagnostic instrument (ref) or WHS items as a symptom scale
Recall period of treatment	12 months (ref) or less
Response rate	Proportion of sample contacted that provided data for the study
Sample size	Total number of study participants
Urbanicity	Information on urban, rural, or mixed setting of study location
MAT	As defined by study; categorized as lenient, moderate, or stringent definition
Disorder severity	As reported by study (mild, moderate, or severe)

MAT, minimally adequate treatment; MDD, major depressive disorder; Ref, reference; WHS, World Health Surveys.

### Statistical analysis

Our primary regression analysis was restricted to data on MDD. We modeled MDD treatment rates as a function of selected covariates listed in Table 2 using a meta-regression: Bayesian, regularized trimmed (MR-BRT) framework to estimate pooled treatment rates adjusted for parameters of interest [24]. This novel meta-analytic modeling approach was developed at the Institute for Health Metrics and Evaluation for modeling data for the GBD study [1]. This approach was used to incorporate between-study heterogeneity in the uncertainty bounds of parameter estimates [24]. This also allowed our methods to adhere to those used within the GBD study. Parameters that contributed to significant differences in treatment rates were retained for meta-analyses reported in the results below. We used fixed effects for selected covariates and random effects for studies chosen a priori to account for between-study variation. Due to considerable heterogeneity and sparsity of data by select covariates, we chose to analyze data on disorder severity, MAT definitions, dysthymia and mood disorders, and treatment rates by year as part of our supplementary analyses (see S1 Appendix, Section 7).

Treatment rates for MDD extracted from selected data sources were used to compute treatment gaps as 1 –Treatment rate. We estimated uncertainty for our analyses at the 1,000 draws level. Estimates of projected treatment gaps were computed using the mean estimate across 1,000 draws, and the 95% UIs are determined on the basis of the 25th and 975th quantile values across a total of 1,000 draws. The generated 95% UI reflected the main sources of sampling uncertainty from both the prevalence and treatment rates. While the MR-BRT analysis incorporated data for all treatment types, we estimated treatment gaps by age, sex, and GBD superregion for health service use and mental health service use, respectively. The estimation of treatment gaps was not undertaken for other treatment types with insufficient data to inform this analysis. GBD prevalence data were combined with treatment rates to calculate the number of treated and untreated cases of MDD. All data analyses and visualizations were performed using R version 4.1 [25].

## Results

### Study characteristics

**Fig 1:**
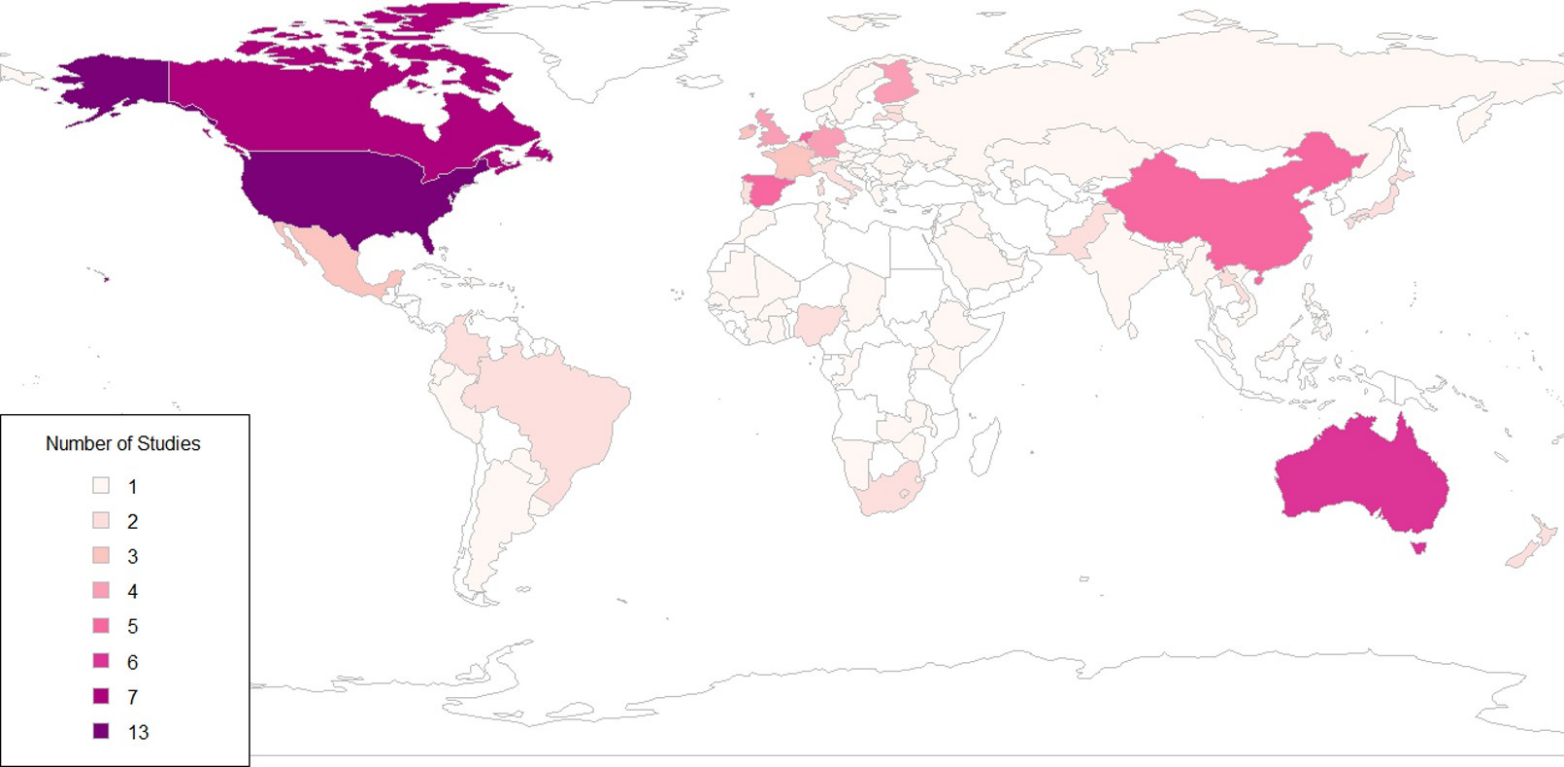
Number of studies on MDD treatment coverage by country. Note: created using open-source software R version 4.1, using the rworldmap package. MDD, major depressive disorder.

We identified 342 data points from 149 studies reporting on treatment rates for MDD from 84 countries. The literature search and data sources are summarized in the appendix (see S1 Appendix, Sections 1–5). Table 3 summarizes the number of available datapoints for each of the 6 treatment types by income group. Fig 1 shows the global availability of relevant studies on treatment rates for MDD.

**Table 3:** Number of datapoints for MDD by treatment type and income group.

Treatment type	Low and lower middle	Upper middle	High-income	Total
Any general health service	1	3	33	37
Any health service	27	20	47	94
Any mental health service	6	14	80	100
Any nonhealth service	1	4	23	28
Any service use	2	14	28	44
MAT	1	9	28	39

MAT, minimally adequate treatment; MDD, major depressive disorder.

### Regression results for MDD data

The coefficients from our regression model are presented in Table 4. Our final model included treatment type, income group, age, sex, and survey instrument as significant predictors of treatment rates. Treatment rates for any service use (β = 0.281 [95% UI: 0.267, 0.294], *p* < 0.001), any health service use (β = 0.172 [95% UI: 0.157, 0.187], *p* < 0.001), and general health service use (β = 0.061 [95% UI: 0.048, 0.073], *p* < 0.001) were significantly higher than those for mental health service use (reference category, β = 0.619 [95% UI: 0.599, 0.639], *p* < 0.001). Treatment rates for MAT (β = −0.116 [95% UI: −0.13, −0.103], *p* < 0.001) and nonhealth service use (β = −0.157 [−95% UI: 0.17, −0.144], *p* < 0.001) were significantly lower than those for mental health service use (reference category). An increase in age was associated with an increase in MDD treatment rates (β = 0.001 [95% UI: 0.0003, 0.001], *p* = 0.002). Treatment rates were higher for females compared to males (β = 0.056 [95% UI: 0.0398, 0.072], *p* < 0.001). The use of a symptom scale (as opposed to diagnostic instruments) was associated with a decrease in treatment rates (β = −0.106 [95% UI: −0.169, −0.042], *p* < 0.001). Treatment rates from upper middle-income locations (β = −0.212 [95% UI: −0.286, −0.138], *p* < 0.001) and low- and lower middle-income locations (β = −0.324 [95% UI: −0.4036, −0.244], *p* < 0.001) were significantly lower than those from high-income locations (reference category).

**Table 4:** Regression coefficients and 95% UIs for MDD treatment rates modeled as a function of select covariates: Treatment type (ref = mental health service use), income group (ref = high-income), age (ref = median approximately 50 years), sex (ref = both), and survey instrument (ref = mental disorder diagnostic instrument).

Covariate	Parameter estimate [95% UI]	*P* value
Intercept^a^	0.619 [0.599, 0.639]	<0.001
Treatment type		
Any service use	0.281 [0.267, 0.294]	<0.001
General health service use	0.061 [0.048, 0.073]	<0.001
MAT	−0.116 [−0.13, −0.103]	<0.001
Health service use	0.172 [0.157, 0.187]	<0.001
Nonhealth service use	−0.157 [−0.17, −0.144]	<0.001
Sample characteristics		
Age	0.001 [0.0003, 0.001]	0.002
Percent female	0.056 [0.0398, 0.072]	<0.001
World Bank Income Group		
Upper middle-income	−0.212 [−0.286, −0.138]	<0.001
Low- and lower middle-income	−0.324 [−0.4036, −0.244]	<0.001
Methodological covariates		
Survey instrument	−0.106 [−0.169, −0.042]	0.001

^a^Intercept represents mental health service use when all other variables are equal to their referent category.

MAT, minimally adequate treatment; MDD, major depressive disorder; Ref, reference; UI, uncertainty interval.

Predicted treatment rates by service type and income group are reported in Table 5. Overall, treatment rates were the highest for any service use, followed by any health service use, and other treatment types. The lowest treatment rates were observed for MAT and nonhealth service use. Pooled MAT treatment rates by stringency and income status are presented in the appendix (see S1 Appendix, Section 7.3). Treatment rates across all service types were the highest for high-income locations compared to upper middle- and low- and lower middle-income locations. For high-income locations, treatment rates ranged from 61% [95% UI: 29%, 89%] for any service use to 19% [95% UI: 1%, 51%] for any nonhealth service use. For upper middle-income locations, treatment rates ranged from 40% [95% UI: 11%, 73%] for any service use to 6% [95% UI: <1%, 30%] for nonhealth service use. For low- and lower middle-income countries, treatment rates ranged from 29% [95% UI: 5%, 63%] for any service use to 1% [95% UI: <1%, 22%] for any nonhealth service use.

**Table 5:** Predicted percentage of MDD cases receiving treatment [95% UI] by income group.

Treatment type	High-income	Upper middle-income	Lower middle- and low-income
Any service use	61 [29, 89]	40 [11, 73]	29 [5, 63]
Health service use	51 [20, 82]	30 [5, 63]	20 [1, 53]
General health service use	39 [12, 72]	20 [1, 51]	12 [<1, 42]
Mental health service use	33 [8, 66]	15 [<1, 45]	8 [<1, 36]
Nonhealth service use	19 [1, 51]	6 [<1, 30]	1 [<1, 22]
MAT	23 [2, 55]	8 [<1, 33]	3 [<1, 25]

Note: 95% UIs incorporate between-study heterogeneity.

MAT, minimally adequate treatment; MDD, major depressive disorder; UI, uncertainty interval.

**Fig 2:**
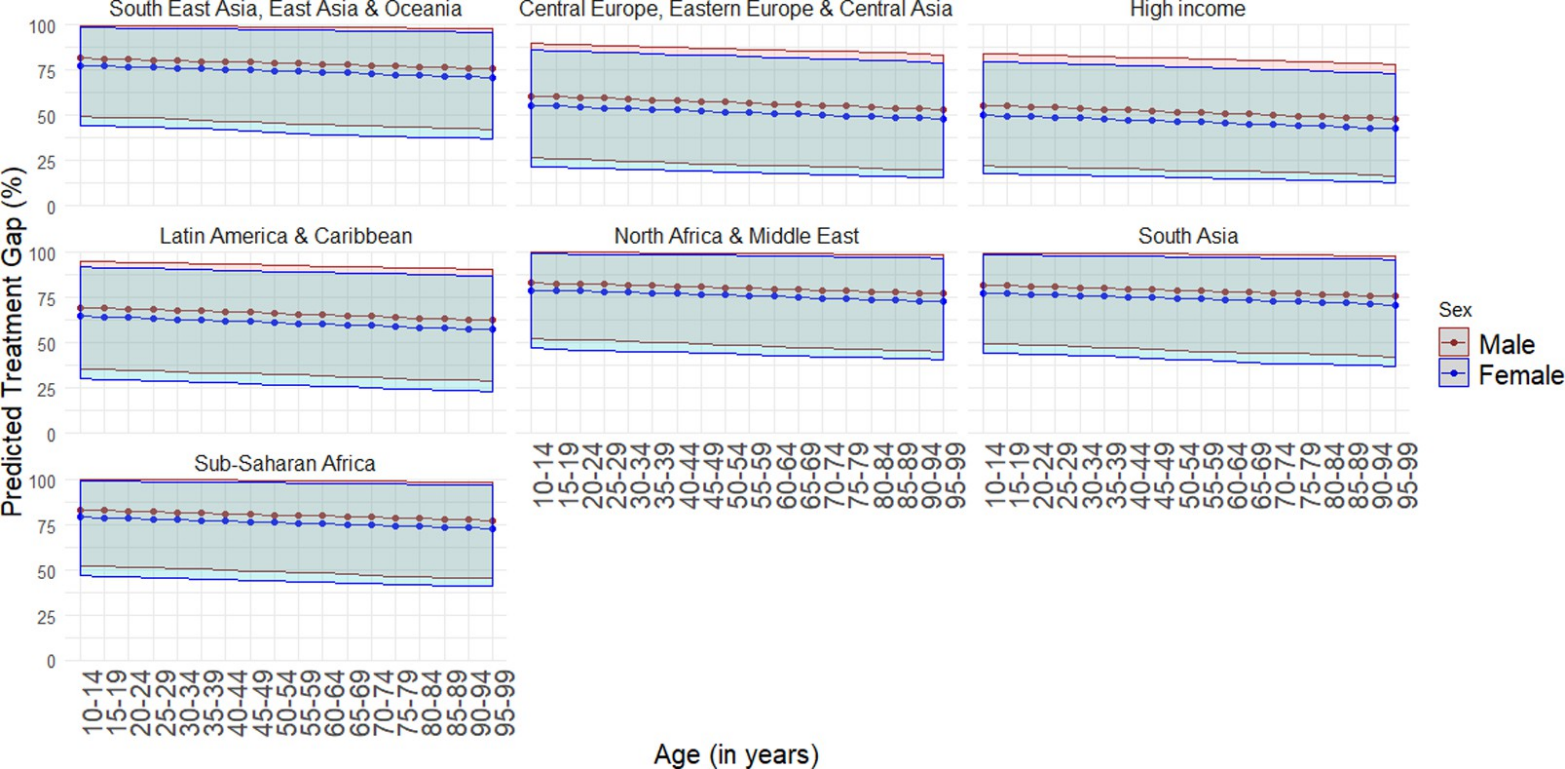
Predicted treatment gap (lines) and 95% UIs (shaded region) for any health service use by age, sex, and GBD superregion. Note: 95% UIs incorporate between-study heterogeneity. GBD, Global Burden of Disease; UI, uncertainty interval.

**Fig 3:**
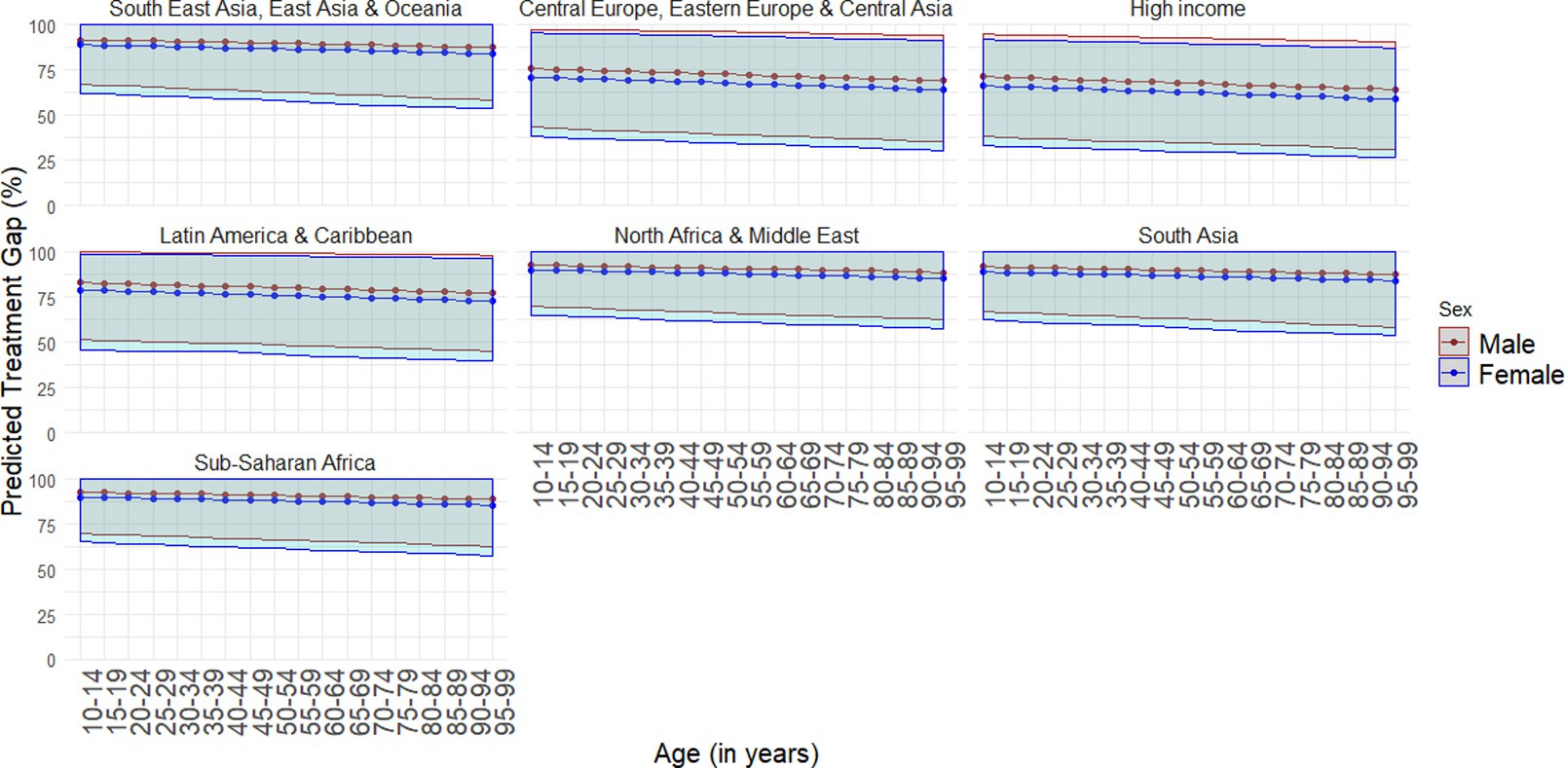
Predicted treatment gap (lines) and 95% UIs (shaded) for any mental health service use by age, sex, and GBD superregion. Note: 95% UIs incorporate between-study heterogeneity. GBD, Global Burden of Disease; UI, uncertainty interval.

We also modeled treatment rates as a function of the covariates described above along with GBD superregion (instead of income group). The regression coefficients from this model are presented in the appendix (see S1 Appendix, Section 6). Table 6 shows overall predicted treatment rates by service type and GBD superregion. Figs 2 and 3 show projected treatment gaps disaggregated by age, sex, and GBD superregion for health service use and mental health service use. They illustrate that the treatment gap was slightly larger for males and decreased with age.

**Table 6:** Predicted percentage of MDD cases receiving treatment [95% UI] by GBD superregion.

Treatment type	Southeast Asia, East Asia, and Oceania/South Asia^a^	Central Europe, Eastern Europe, and Central Asia	High-income	Latin America and Caribbean	North Africa and Middle East/sub-Saharan Africa^a^
Any service use	30 [6, 63]	55 [23, 84]	61 [30, 88]	45 [16, 77]	28 [5, 60]
Health service use	21 [2, 53]	44 [15, 76]	50 [21, 80]	34 [9, 68]	19 [1, 49]
General health service use	12 [<1, 41]	33 [8, 65]	39 [12, 70]	24 [4, 57]	11 [<1, 39]
Mental health service use	8 [<1, 35]	28 [5, 59]	33 [8, 64]	19 [2, 50]	7 [<1, 32]
Nonhealth service use	2 [<1, 21]	15 [<1, 44]	19 [2, 48]	8 [<1, 35]	1 [<1, 19]
MAT	3 [<1, 25]	18 [1, 48]	22 [3, 52]	11 [<1, 39]	2 [<1, 22]

Note: 95% UIs incorporate between-study heterogeneity.

^a^GBD superregions combined within model due to limited number of estimates informing these regions.

GBD, Global Burden of Disease; MAT, minimally adequate treatment; MDD, major depressive disorder; UI, uncertainty interval.

## Discussion

Our systematic review identified 342 datapoints from 84 countries on the treatment rates of MDD. From this dataset, we characterized the patterns of service use for MDD. Treatment rates were modeled as a function of service type, location, age, sex, and survey instrument. Treatment rates for any service use, any health service use, and any general health service use were significantly higher than mental health service use. Although mental health service use is traditionally considered the most ideal for MDD, the higher treatment rates of broader categories of any, health, or general health service use indicates the importance of these types of services used to treat MDD given the lack of specialized mental health services in many countries. These findings are also largely consistent with WHO recommendations on treatment of mental disorders within general or primary healthcare settings for all countries [7].

Age was associated with an increase in treatment rates. This is consistent with earlier findings that older age is typically associated with greater use of treatment services. Treatment rates were higher in females than males. This is also consistent with trends found in other studies that females are perhaps more likely to detect and seek out care for emotional problems than males [16]. The type of survey instrument used was important to incorporate in our main model as methodological features that may impact population-based analyses. We accounted for differences in treatment rates between studies using a diagnostic instrument and our WHS estimates derived by a symptom scale and which were adjusted prior to analyses.

Low- and middle-income countries had significantly lower treatment rates compared to high-income locations. With up to 75% of individuals with MDD residing in low- and middle-income countries, this indicates that a substantial proportion of people with MDD globally do not access any health-related services. Our findings echo the importance of existing calls for the prioritization of mental health in national health agendas. Many countries featured in our review still lack the mental health policy, legislation, or resources to guide their mental health programs and services [7,26]. Our findings also showed that even in high-income countries where treatment rates are comparatively higher, the majority of individuals receiving care for MDD failed to receive a level of care that is consistent with practice guideline recommendations. Only a small minority of individuals with MDD accessed treatment in the specialized mental healthcare system or received MAT.

In this paper, we analyzed rates of MAT by categorizing study-reported definitions of MAT as either “stringent,” i.e., requiring some combination of at least 8 visits to a mental health professional and at least 30 days of prescribed antidepressant use or “nonstringent,” i.e., having lower threshold for mental health visits and medication use, and deemed additional types of service use as adequate treatment. Stringent definitions of MAT were most commonly found in studies originating from high-income countries (e.g., Canada, Finland, USA, and Spain). Treatment rates for stringent definitions of MAT were lower than those for nonstringent definitions of MAT (see S1 Appendix, Section 7.3). It is plausible that stringent MAT may be challenging to attain in many countries especially if mental health treatment is provided within primary or general healthcare settings by trained healthcare providers instead of specialized mental health professionals [6]. In countries where attaining MAT may pose a challenge, alternative interventions that leverage community and nonspecialized human resources and ensure appropriate levels of treatment intensity have shown promise [27–30].

The findings presented here and elsewhere indicate that access to care for MDD needs to be improved. However, the specifics of which components of care increase “access” still need to be systematically explored. Dedicated mental health services, institutes, and hospital units along with community-based care exist in many high-income countries. However, mental healthcare institutions in low- and middle-income countries are likely underresourced and overburdened with treatment for competing, acute health conditions. However, simply access alone is not enough. The proportion of people who receive sufficient care once they enter treatment is still difficult to estimate and is unclear from the current literature. MAT is also difficult to quantify because not everyone who meets criteria for MDD will need or want care. Therefore, it is important to consider not simply the presence of services, but what behavioral or environmental drivers impact contact with and adherence to treatment. A recent paper estimated a 90% gap in effective treatment with lack of utilization and inadequate quality or adherence being critical bottlenecks [13]. While this provides an important decomposition of elements of treatment coverage and quality, it is also important to consider variation in real-world treatment settings and variation in MAT thresholds that impact treatment rates as shown in this analysis. Treatment gaps for MDD also need to consider gaps in psychosocial and physical healthcare [31]. Psychosocial interventions have been shown to be highly effective in symptom reduction, and physical healthcare is important to include considering the high and often untreated physical comorbidity and premature mortality that accompanies MDD and other mental disorders [32].

Efforts to close the depression treatment gap would also need significant boosts in funding allocations. Global health financing has historically been prioritized for malaria, HIV/AIDs, and tuberculosis—which are some of the leading causes of disability and mortality in many low- and middle-income countries. However, financing for mental health is still far from adequate. In 2019, development assistance for health (DAH) for noncommunicable diseases (which includes mental disorders) for Sustainable Development Goal (SDG) 3 targets was $0.7 billion for 135 low- and middle-income countries—which is less than 2% of the total estimated DAH in 2019 of $40.6 billion [33]. Therefore, it is important to align funding priorities with epidemiological shifts in countries that are likely to be accompanied by an increase in noncommunicable disease burden including mental disorders. A global return on investment analysis by Chisholm and colleagues showed that scaling up effective treatment for depression and anxiety disorders leads to 43 million extra years of healthy life and a net present economic value of $310 billion between 2016 to 2030 [3]. The Comprehensive Mental Health Action Plan 2013–2020 adopted by the World Health Assembly was recently revised and extended through 2030 to include an updated set of indicators. Of particular significance is a newly added indicator to quantify the proportion of people with depression who are using services during the past 12 months [7,8,26]. The presence of an indicator to track treatment use among those with depression specifically may serve as an important impetus for regular data monitoring and tracking for treatment coverage. In the United Nations SDGs, mental health was for the first time explicitly recognized within the concept of Universal Health Coverage [34]. It is clear that providing effective services for people with depression, integrated into general health services, care for HIV or maternal and child health, is a vital element of basic healthcare provisions [35,36]. As we now have evidence for effective and feasible interventions suitable for low-, middle-, and high-income countries, we call upon national and international organizations to make firm and time-bound commitments to make adequate resources available for scaling up the provision of mental health services so that “no one is left behind.” This is particularly pertinent during the current COVID-19 pandemic, which has been accompanied by an increase in the prevalence of depression and a simultaneous decrease in access to services in many countries [37].

The analyses conducted were limited by lack of high-quality data on service use. Most studies originate from high-income countries largely located in North America and Western Europe. However, low- and middle-income countries (mostly in sub-Saharan Africa and South Asia) that comprise approximately 70% of the world’s population and 80.9% of MDD nonfatal disability globally accounted for only 22% of studies on MDD in this dataset. Despite the significant disparities in available data, the available evidence indicates that the treatment gap for MDD is consistently wide across most locations. There was considerable variation in treatment rates across countries, suggesting that resources available for MDD not only continue to be scarce but unequally distributed across the globe, and far from commensurate to the prevalence of MDD [38].

Some additional limitations are important to note. First, there are gaps in the available data, which should be recognized. Only 22% or less than a quarter of the studies originated from low- or lower middle-income countries. Additionally, most of the available data represented treatment rates for any health service or mental health service accessed, with fewer studies reporting on access to other types of services especially in low- or lower middle-income countries. Both limitations restricted the generalizability of our findings. This is reflected in the large uncertainty bounds accompanying estimates for low- and lower middle-income countries, which should be interpreted with caution. Second, given the nature of our systematic review, we had to rely on definitions for service use set by each individual data source. Definitions for what comprise MAT in particular varied widely by data source and highlighted the lack of consistency in the literature in how this concept should be defined. Thornicroft and colleagues restricted their definition to those “receiving either pharmacotherapy (at least 1 month of a medication and 4 visits to any type of medical doctor) or psychotherapy (at least 8 visits with any professional including religious or spiritual advisor, social worker, or counselor),” but the extent to which this should be considered a practice guideline recommendations is unclear as depression exists on a severity continuum with more intensive treatment needed for depression of higher severity. Third, there were insufficient data by location and year to appropriately analyze changes in MDD treatment rates over time for all locations (see S1 Appendix, Section 7). Fourth, there were limited data on treatment rates disaggregated by sex and age, which may have resulted in small parameter estimates for these variables.

This study sets a methodological framework from which new data on this topic may be analyzed in the future. We improve upon earlier work by applying updated modeling methods that better capture heterogeneity in the data and account for bias that may be contributed by study-level characteristics. In doing so, we highlighted various literature gaps and methodological considerations for researchers undertaking new mental health surveys in the future. Findings from this study may also contribute to future work in modeling potentially avoidable burden of MDD in varying scenarios of treatment coverage.

## Conclusions

Our findings suggest that treatment coverage for MDD continues to be low globally and, in particular, in low- and lower middle-income countries. Higher treatment rates of broader categories of any, health, or general health service use indicated the importance of these types of services to treat MDD given the lack of specialized mental health services in many countries, particularly those that are resource poor. However, even in high-income countries where treatment rates are comparatively higher, many individuals failed to receive a level of care consistent with practice guideline recommendations. Ultimately, our findings emphasize the need for governments and policy makers to reconsider the availability of appropriate care for MDD and facilitators of treatment as they respond to the large burden imposed by this disorder.

## Supporting information

S1 Appendix Additional information on study methods and findings. (DOCX)

S1 Checklist Preferred Reporting Items for Systematic Reviews and Meta-Analyses (PRISMA) checklist. (DOCX)

## Abbreviations

DAH: development assistance for health
DALY: disability-adjusted life year
GBD 2019: Global Burden of Disease Study 2019
MAT: minimally adequate treatment
MDD: major depressive disorder
MDE: major depressive episode
MR-BRT: meta-regression: Bayesian, regularized trimmed
SDG: Sustainable Development Goal
UI: uncertainty interval
WHS: World Health Surveys
