# Supplementary material for: The global gap in treatment coverage for major depressive disorder in 84 countries from 2000–2019: A systematic review and Bayesian meta-regression analysis
Source: PLoS Med. 2022 Feb 15;19(2):e1003901. doi: 10.1371/journal.pmed.1003901 (PMC8846511; doi:10.1371/journal.pmed.1003901)
Supplement: S1 Appendix — (DOCX) [file pmed.1003901.s001.docx]

**Table of Contents**

1. Systematic Review Search Strings
2. PRISMA flowchart for MDD treatment coverage studies
3. World Health Surveys Data Sources
4. MDD Treatment Coverage Data sources
5. Study Characteristics
6. Meta-regression Coefficients (By Super-region)
7. Supplementary Results:
   1. Treatment Rates by MDD severity
   2. Treatment Rates by Year
   3. Minimally Adequate Treatment (MAT) rates
   4. Treatment Rates for Dysthymia, Depressive Disorders, and Mood Disorders
   5. Funnel plots

______________________________________________________________________________________________________________________

1. **Systematic Review Search Strings:**

PubMed:

Treatment rates systematic review:

- ((((“Mental Health Services” AND “Mental Disorders” AND (Epidemiology OR “Health Care Surveys”) )OR (((((("Mental Health Services/ utilization" OR “unmet needs” OR Untreated OR (Treatment AND rate*) OR (Treatment AND gap)) AND (epidemiolog* OR population*) ) AND ( ("Mental health" OR "Mental disorder*" OR " Mental illness*" OR "Psychiatric disorder*" OR "Psychiatric illness*") OR ("Depress*" OR "Dysthymi*" OR "Mood disorder*" OR "Affective disorder*") OR Bipolar OR Schizophrenia OR (("Substance" OR "Addictive" OR "Addiction" OR "Drug dependence" OR "Cannabi*" OR "Hallucinogen*" OR "Opioid*" OR "Heroin*" OR "Amphetamine*" OR "Cocaine*" OR "Marijuana*") AND ("Use" OR "Dependence" OR "disorder")) OR ( "Anxiety" OR "Obsessive compulsive" OR "Agoraphobia" OR "Social phobia" OR "Phobia" OR "Post-traumatic stress" OR "Posttraumatic stress" OR "PTSD" OR "Panic disorder"))))) AND "humans"[Filter]) NOT "clinical trial"[Filter]) NOT ((cancer[sb] OR AIDS[sb])))

Embase:

- (major AND depression:ab,ti OR depression:ab,ti OR depres*:ab,ti OR dysthymia:ab,ti OR mental:ab,ti OR behavioral:ab,ti) AND ((mental AND health AND service:ab,ti OR health) AND service:ab,ti OR care:ab,ti OR service) AND utilization:ab,ti

Prevalence systematic review:

- (((((((((((((((((depress*[Title/Abstract]) OR dysthymi*[Title/Abstract]) OR bipolar[Title/Abstract]) OR manic[Title/Abstract]) OR mania[Title/Abstract]) OR "mood disorders"[Title/Abstract]) OR "mood disorder"[Title/Abstract]) OR mood disorders[MeSH Terms]) OR depressive disorders[MeSH Terms]) OR depressive disorders, major[MeSH Terms]) OR bipolar disorders[MeSH Terms]) OR dysthymic disorders[MeSH Terms] AND ((((((((((((((((prevalen*[Title/Abstract]) OR mortality[Title/Abstract]) OR death*[Title/Abstract]) OR inciden*[Title/Abstract]) OR recurren*[Title/Abstract]) OR remission[Title/Abstract]) OR duration[Title/Abstract]) OR remit*[Title/Abstract]) OR epidemiolog*[Title/Abstract]) OR prevalence[MeSH Terms]) OR mortality[MeSH Terms]) OR incidence[MeSH Terms]) OR recurrence[MeSH Terms])

1. **PRISMA flowchart for MDD treatment coverage studies:**

Additional records identified through other sources
(n = 170)

Records identified through database searching
(n = 31,267)

Records excluded
(n = 28,737)

Records screened after duplicates removed
(n = 30,109)

Records Screened (Title and Abstract)
(n = 30,109)

Full-text articles excluded
(n = 1,221)

Reasons for exclusion

Sample not representative of general population: 319

Year of data collection <2000: 106

Definition of mental disorder use not suitable: 102

Lifetime rates of service use reported without point prevalence: 56

No data in sample with depressive disorders: 582

Data already captured in another included study: 14

No access to data: 1

Not a peer-reviewed paper: 41

Full-text articles assessed for eligibility
(n = 1,372)

Full-text articles included in quantitative synthesis
(n = 151, of which 149 report on major depressive disorder)

1. **World Health Survey data sources:**

The World Health Surveys (WHS) were implemented by WHO to monitor adult health outcomes and health systems between 2002 and 2004 in countries chosen to represent all world regions. Samples were representative of the general population and probabilistically selected. For each country, unit record data were aggregated to estimate the proportion of cases likely meeting diagnostic criteria for MDD and the proportion of these probable MDD cases accessing any health service in the last two weeks. The survey item relating to service use identified individuals taking medication or receiving other professional treatment for their symptoms of depression in the last two weeks. Treatment was defined as attending psychological therapy or counselling session with a trained health professional during the last two weeks. The survey items relating to depression in the WHS captured the majority but not all symptoms required for a full-diagnosis of MDD according to the DSM or ICD. As such, the estimated treatment rates likely pertained to a combination of individuals with MDD and sub-threshold MDD.

We conducted a sensitivity analysis to examine the difference between other treatment rates available in our dataset where MDD was defined according to full DSM/ICD criteria and those derived from the WHS. There were three countries (Australia, Spain, France) for which we had comparable WHS and diagnostic based treatment rates for ‘any health service’. The pooled ratio of other diagnostic-based treatment rates: WHS treatment rates was 1.4 (95% confidence interval: 1.3-1.6). This suggested that surveys capturing a full diagnosis of MDD produced treatment rates that were on average 1.4 times higher than WHS surveys. We maximized data inclusion by including the WHS treatment rates in our estimation of treatment gaps. To improve comparability of WHS treatment rates with other treatment rates in the dataset, WHS estimates were adjusted upwards towards the level they would have been had they been based on a full-diagnosis of MDD using our estimated ratio. The diagnostic algorithm used to identify probable cases of MDD within the WHS, the analysis to estimate the pooled ratio of other diagnostic-based treatment rates: WHS treatment rates, and a comparison of the adjusted and unadjusted WHS estimates are shown below.

Table S1 summarizes the diagnostic algorithm used to identify probable cases of major depressive disorder within each World Health Survey (WHS).

**Table S1: DSM-IV-TR criteria for major depressive disorder with corresponding World Health Survey item**

| DSM-IV-TR criteria for major depressive disorder (single episode) | WHS Item | WHS item description |
| --- | --- | --- |
| First determine if all of the following apply; | | |
| Not a mixed episode (e.g. bipolar disorder) | N/A | N/A |
| Symptoms cause clinically significant distress or impairment in social, occupational or other important areas of concern | N/A | N/A |
| Not due to direct effect of a substance | N/A | N/A |
| Not accounted for by bereavement unless continuous for over 2 months or severe functional impairment, morbid preoccupation with worthlessness, psychotic symptoms or psychomotor retardation | N/A | N/A |
| If all of the above is true, must have one or both of these symptoms; | | |
| Present for the same 2 week period | Q6031 | *“Was this period [of sadness/loss of interest/low energy] for more than 2 weeks?”* |
| Depressed mood most of the day and nearly every day, self-reported or observed by others | Q6028 | *“Have you had a period lasting several days when you felt sad, empty or depressed?”* |
| Or | | |
| Markedly diminished interest or pleasure in all, or almost all, activities on most days, self-reported or reported by others | Q6029 | “*Have you had a period lasting several days when you lost interest in most things you usually enjoy such as hobbies, personal relationships or work?”* |
| Must have either one or both of the above symptoms plus 3 or 4 of these to make a total of 5 or more symptoms; | | |
| Significant weight loss (not due to dieting) or gain (e.g. 5% change in one month); or decrease or increase in appetite nearly every day | Q6033 | *“During this period, did you lose your appetite?”* |
| Insomnia or hypersomnia nearly every day | Q2080 | *“Overall in the last 30 days, how much of a problem did you have with sleeping, such as falling asleep, waking up frequently during the night or waking up too early in the morning?”* |
| Psychomotor agitation or retardation nearly every day, observable by others | Q6034 | *“During this period, did you notice any slowing down in your thinking?”* |
| Fatigue or loss of energy nearly every day | Q6030 | “*Have you had a period lasting several days when you have been feeling your energy decreased or that you are tired all the time?”* |
| Feelings of worthlessness or excessive or inappropriate guilt nearly every day; May be delusional; Not merely self-reproach or guilt about being sick | N/A | N/A |
| Diminished ability to think or concentrate, or indecisiveness, nearly every day (self-reported or observed by others) | Q2050 | *“Overall in the last 30 days, how much difficulty did you have with concentrating or remembering things?”* |
| Recurrent thoughts of death, recurrent suicidal ideation without a specific plan, or a suicide attempt or a specific plan for committing suicide |  |  |

*Note: DSM-IV-TR: Diagnostic and Statistical Manual of Mental Disorders Fourth Edition, Test Revision; WHS: World Health Survey*

A participant was counted as a probable case if major depressive disorder if Q6031 = 1 AND ((Min (Q6028, Q6029) = 1) AND Min (Q6033, Q2080 Q6034, Q6030, Q2050) =>4) OR (Min (Q6028, Q6029) = 2) AND Min (Q6033, Q2080, Q6034, Q6030, Q2050) =>3))

- For Q6028, Q6029, Q6030, Q6031, Q6033, and Q6034, a value of 5 (“No”) was recoded as 0.
- For Q6025 and Q6028, a value =>3 was recoded as 1, and a value < 3 was recoded as 0.

The WHS item related to any health service utilization asked the following question;

“*Have you taken medication or received other professional treatment for your depression symptoms? The purpose of this question is to find out if the respondent is currently under treatment for depression. An answer of “yes” is appropriate only if the respondent was taking any medications for depression during the last two weeks, or received some kind of treatment such as attending a psychological therapy or counselling session (often referred to as “therapy” or “psychotherapy”) with a trained health professional during the last two weeks”*

There were three countries (Australia, Spain, France) for which we had comparable WHS and diagnostic based treatment rates for ‘any health service’. These are summarised in Table S2. The pooled ratio of other diagnostic-based treatment rate: WHS treatment rates was 1.4 (95% confidence interval: 1.3-1.6).

**Table S2: Comparable World Health Survey and diagnostic based treatment rates for ‘any health service’ in the dataset**

| **WHS data-source** | | | | | | **Corresponding diagnostic data-source** | | | |
| --- | --- | --- | --- | --- | --- | --- | --- | --- | --- |
| **Country** | **Sex** | **Year** | **Age range** | **Treatment rate** | **Source** | **Year** | **Age range** | **Treatment rate** | **Source** |
| Australia | F | 2003-2003 | 18-100 | 34.8% | (1) | 2007-2007 | 16-85 | 62.2% | (1) |
| Australia | M | 2003-2003 | 18-100 | 36.6% | (2) | 2007-2007 | 16-85 | 42.9% | (3) |
| Spain | F | 2002-2003 | 18-96 | 48.8% | (4) | 2001-2002 | 18-100 | 60.8% | (5) |
| Spain | M | 2002-2003 | 18-96 | 35.8% | (6) | 2001-2002 | 18-100 | 54.2% | (7) |
| France | P | 2003-2003 | 18-93 | 40.8% | (8) | 2005-2005 | 20-100 | 60.4% | (9) |

*Note: WHS: World Health Survey; P: Person; F: Female; M: Male*

The estimated pooled ratio suggested that surveys capturing a full diagnosis of MDD produced treatment rates that were approximately 1.4 times higher than WHS surveys. We maximized data inclusion by including the WHS treatment rates in our estimation of treatment gaps and adjusting them upwards towards the level they would have been had they been based on a full-diagnosis of MDD using our estimated pooled ratio. The adjusted and un-adjusted WHS estimates are summarized in Table S3.

**Table S3: Adjusted and un-adjusted WHS treatment rates and gaps for major depressive disorder**

|  | | | **Un-adjusted estimates** | | **Adjusted estimates** | |
| --- | --- | --- | --- | --- | --- | --- |
| **Country** | **Year** | **Age range** | **Treatment rate % (standard error)** | **Treatment gap %**  **(95% confidence interval)** | **Treatment rate % (standard error)** | **Treatment gap % (95% confidence interval)** |
| Australia | 2003-2003 | 18-100 | 35.4 (3.6) | 64.6 (57.2-71.7) | 50.2 (5.8) | 49.8 (38.4-60.9) |
| Austria | 2003-2003 | 20-93 | 38.1 (10.6) | 61.9 (40.4-82.1) | 54 (15.3) | 46 (18.5-75.7) |
| Bangladesh | 2003-2003 | 18-100 | 2.6 (0.6) | 97.4 (96.1-98.4) | 3.7 (0.9) | 96.3 (94.3-97.9) |
| Belgium | 2003-2003 | 17-100 | 37.1 (5.8) | 62.9 (50.8-74.2) | 52.7 (8.7) | 47.3 (31.1-65) |
| Bosnia and Herzegovina | 2003-2003 | 18-85 | 35 (5.6) | 65 (53.5-75.3) | 49.6 (8.4) | 50.4 (34.2-66.8) |
| Brazil | 2003-2003 | 18-96 | 25.9 (1.7) | 74.1 (70.6-77.4) | 36.8 (3.1) | 63.2 (57.2-69.3) |
| Burkina Faso | 2002-2003 | 18-100 | 8.4 (1.7) | 91.6 (87.9-94.8) | 11.9 (2.5) | 88.1 (82.9-92.5) |
| Chad | 2003-2003 | 18-100 | 6 (1.2) | 94 (91.4-96.1) | 8.6 (1.8) | 91.4 (87.8-94.5) |
| China | 2002-2002 | 18-96 | 1 (1.2) | 99 (95.7-100) | 1.5 (1.7) | 98.5 (94.1-100) |
| Comoros | 2003-2003 | 16-86 | 8.3 (2.7) | 91.7 (86.1-96.2) | 11.8 (3.9) | 88.2 (79.9-94.7) |
| Cote d'Ivoire | 2003-2003 | 18-96 | 8.7 (2.6) | 91.3 (85.6-95.9) | 12.3 (3.8) | 87.7 (79.1-94.2) |
| Croatia | 2003-2003 | 18-94 | 44.8 (6.3) | 55.2 (42.7-67.6) | 63.5 (9.6) | 36.5 (18.3-56.9) |
| Czech Republic | 2002-2003 | 18-95 | 10.6 (4.5) | 89.4 (78.6-96.5) | 15.1 (6.4) | 84.9 (70.3-95.1) |
| Denmark | 2003-2003 | 17-93 | 53.1 (8.8) | 46.9 (30.3-64) | 75.3 (13.2) | 24.7 (5.1-53.8) |
| Dominican Republic | 2003-2003 | 18-100 | 4 (1.1) | 96 (93.5-97.9) | 5.7 (1.6) | 94.3 (90.8-96.9) |
| Ecuador | 2003-2003 | 15-100 | 16.3 (2.5) | 83.7 (78.4-88.4) | 23.1 (3.8) | 76.9 (69.2-83.9) |
| Estonia | 2003-2003 | 17-93 | 14.6 (4.4) | 85.4 (76.3-93) | 20.6 (6.3) | 79.4 (66.2-90.2) |
| France | 2003-2003 | 18-93 | 40.8 (5.1) | 59.2 (49.5-69) | 57.8 (7.9) | 42.2 (27.2-58.6) |
| Georgia | 2003-2003 | 18-100 | 13.4 (2.5) | 86.6 (81.3-91.3) | 19 (3.6) | 81 (73.6-87.8) |
| Germany | 2004-2004 | 18-95 | 54.5 (8.7) | 45.5 (28.7-62.9) | 77.3 (13) | 22.7 (3.7-52.4) |
| Ghana | 2003-2003 | 18-100 | 2.8 (1) | 97.2 (95-98.8) | 4 (1.4) | 96 (93-98.3) |
| Greece | 2003-2003 | 18-96 | 11 (3.7) | 89 (81-95.1) | 15.5 (5.3) | 84.5 (73.3-92.9) |
| India | 2003-2003 | 18-100 | 4.7 (0.7) | 95.3 (93.8-96.6) | 6.6 (1.1) | 93.4 (91.1-95.4) |
| Ireland | 2003-2003 | 18-93 | 42.4 (8.7) | 57.6 (41-74.3) | 60.2 (12.8) | 39.8 (15.9-65.6) |
| Italy | 2003-2003 | 18-97 | 39.6 (6.7) | 60.4 (46.8-72.8) | 56.2 (10) | 43.8 (24.9-64.4) |
| Kazakhstan | 2002-2003 | 18-90 | 5.5 (1.9) | 94.5 (90.3-97.7) | 7.8 (2.7) | 92.2 (86.2-96.5) |
| Kenya | 2004-2004 | 18-100 | 4.3 (1.2) | 95.7 (93.2-97.7) | 6.1 (1.7) | 93.9 (90.3-96.7) |
| Laos | 2003-2003 | 18-100 | 23.6 (5.4) | 76.4 (65.1-86.1) | 33.4 (7.9) | 66.6 (51-81.1) |
| Latvia | 2003-2003 | 18-90 | 32.4 (6.6) | 67.6 (54.1-79.3) | 45.9 (9.7) | 54.1 (35.6-73.3) |
| Luxembourg | 2003-2003 | 18-91 | 34.7 (7.7) | 65.3 (49.2-79.2) | 49.3 (11.3) | 50.7 (30-71.6) |
| Malawi | 2003-2003 | 18-100 | 0 | 100 | 0 | 100 |
| Malaysia | 2003-2003 | 18-100 | 8.9 (2.8) | 91.1 (85.2-95.6) | 12.6 (4) | 87.4 (79-94.2) |
| Mali | 2003-2003 | 15-100 | 4.8 (1.9) | 95.2 (91.2-98.1) | 6.7 (2.6) | 93.3 (87.3-97.3) |
| Mauritania | 2003-2003 | 18-100 | 24.9 (3.6) | 75.1 (67.6-82.1) | 35.4 (5.5) | 64.6 (53.6-74.7) |
| Mauritius | 2003-2003 | 18-94 | 22.5 (2.3) | 77.5 (72.4-81.9) | 31.9 (3.7) | 68.1 (60.8-75.2) |
| Morocco | 2003-2003 | 18-100 | 5.6 (0.8) | 94.4 (92.7-95.9) | 7.9 (1.2) | 92.1 (89.5-94.2) |
| Myanmar | 2003-2003 | 18-100 | 7.3 (3.9) | 92.7 (83.1-98.4) | 10.4 (5.5) | 89.6 (76.2-97.9) |
| Namibia | 2003-2003 | 18-100 | 19.8 (3.1) | 80.2 (73.9-86.2) | 28.1 (4.6) | 71.9 (62.4-80.2) |
| Netherlands | 2004-2004 | 18-85 | 26.1 (4.7) | 73.9 (64.4-82.4) | 37.1 (6.9) | 62.9 (49.5-75.8) |
| Norway | 2003-2003 | 18-94 | 21.5 (6.4) | 78.5 (64.3-89.4) | 30.5 (9.2) | 69.5 (50.6-85.7) |
| Pakistan | 2003-2004 | 18-100 | 9.9 (1.3) | 90.1 (87.2-92.5) | 14 (2) | 86 (81.9-89.9) |
| Paraguay | 2002-2003 | 18-96 | 12 (2) | 88 (83.8-91.6) | 17 (2.9) | 83 (76.7-88.3) |
| Philippines | 2003-2003 | 18-100 | 8.7 (1.6) | 91.3 (87.9-94.1) | 12.4 (2.4) | 87.6 (82.6-91.9) |
| Portugal | 2003-2003 | 18-95 | 53.6 (3.9) | 46.4 (39-54.5) | 76 (6.9) | 24 (11.7-39.6) |
| Republic of Congo | 2003-2003 | 16-86 | 28.2 (2.6) | 71.8 (66.5-76.7) | 39.9 (4.3) | 60.1 (51.6-68.3) |
| Russia | 2003-2003 | 18-95 | 10.4 (1.9) | 89.6 (85.6-93.1) | 14.7 (2.8) | 85.3 (79.3-90.1) |
| Senegal | 2003-2003 | 18-100 | 7 (2.6) | 93 (87.2-97.1) | 9.9 (3.7) | 90.1 (81.8-96.2) |
| Slovakia | 2003-2003 | 18-100 | 5.9 (3) | 94.1 (86.9-98.4) | 8.4 (4.3) | 91.6 (82.4-97.7) |
| Slovenia | 2003-2003 | 18-92 | 18.8 (6.9) | 81.2 (66.7-92.9) | 26.6 (9.9) | 73.4 (52-90.3) |
| South Africa | 2002-2003 | 18-100 | 15.1 (4.1) | 84.9 (76.1-91.6) | 21.4 (5.9) | 78.6 (66.1-88.4) |
| Spain | 2002-2003 | 18-96 | 45.8 (2.3) | 54.2 (49.7-58.5) | 65 (4.8) | 35 (26-44.6) |
| Sri Lanka | 2003-2003 | 18-100 | 6.9 (2.2) | 93.1 (88.1-96.7) | 9.8 (3.2) | 90.2 (83.2-95.5) |
| Swaziland | 2003-2003 | 18-100 | 32.5 (3.4) | 67.5 (60.9-74.2) | 46 (5.5) | 54 (43.5-64.6) |
| Sweden | 2003-2003 | 18-92 | 23.1 (4.9) | 76.9 (66.9-85.7) | 32.8 (7.2) | 67.2 (53.3-80.8) |
| Tunisia | 2003-2003 | 18-100 | 10.6 (1.5) | 89.4 (86.1-92.3) | 15.1 (2.3) | 84.9 (80.2-89.3) |
| Ukraine | 2002-2003 | 18-96 | 9.5 (2.2) | 90.5 (85.4-94.3) | 13.5 (3.3) | 86.5 (79.7-92) |
| United Arab Emirates | 2003-2003 | 18-91 | 3.1 (2.2) | 96.9 (91.1-99.7) | 4.4 (3.1) | 95.6 (88.1-99.5) |
| United Kingdom | 2004-2004 | 18-100 | 41.2 (5.3) | 58.8 (48.5-69.2) | 58.4 (8.2) | 41.6 (26.3-58.2) |
| Uruguay | 2002-2003 | 18-100 | 25.3 (4.2) | 74.7 (65.6-82.2) | 35.9 (6.3) | 64.1 (52.3-75.9) |
| Vietnam | 2002-2003 | 18-96 | 12.6 (8.3) | 87.4 (66.9-98.3) | 17.9 (11.8) | 82.1 (54.1-97.9) |
| Zambia | 2003-2003 | 18-100 | 5.1 (1.5) | 94.9 (91.3-97.5) | 7.3 (2.2) | 92.7 (87.9-96.3) |
| Zimbabwe | 2003-2003 | 18-100 | 5.4 (2.2) | 94.6 (89.9-98.2) | 7.6 (3.1) | 92.4 (85.1-97.4) |

1. **MDD Treatment Coverage Data sources:**

Table S4: List of Data sources and MDD Treatment Coverage Estimates

| **Data Source** | **Treatment Type** | **Treatment Coverage Estimate (%)** | **95% Lower Uncertainty Interval** | **95% Upper Uncertainty Interval** |
| --- | --- | --- | --- | --- |
| (10) | Any mental health service | 32.9% | 29.8% | 36.0% |
| (10) | Any general health service | 32.5% | 28.0% | 37.0% |
| (10) | Any health service | 51.7% | 47.4% | 56.0% |
| (10) | Any non-health service | 16.8% | 13.5% | 20.1% |
| (10) | Any service use | 56.8% | 52.5% | 61.1% |
| (10) | MAT - stringent | 17.1% | 14.2% | 20.1% |
| (10) | MAT - stringent | 4.8% | 3.2% | 6.5% |
| (10) | MAT - stringent | 19.6% | 16.5% | 22.8% |
| (10) | MAT - stringent | 2.4% | 1.2% | 3.5% |
| (10) | MAT - stringent | 21.3% | 18.1% | 24.5% |
| (11) | Any service use | 59.3% | 55.4% | 63.0% |
| (12) | Any service use | 34.1% | 29.1% | 39.2% |
| (12) | Any general health service | 24.2% | 19.7% | 28.7% |
| (12) | Any mental health service | 13.2% | 3.7% | 22.6% |
| (13) | Any service use | 33.0% | 28.9% | 37.1% |
| (14) | Any mental health service | 62.4% | 61.2% | 63.6% |
| (15) | Any mental health service | 25.2% | 12.5% | 37.9% |
| (15) | Any general health service | 8.9% | 0.0% | 19.1% |
| (15) | Any health service | 27.1% | 12.8% | 41.4% |
| (15) | Any non-health service | 14.6% | 0.3% | 28.9% |
| (15) | Any service use | 33.8% | 17.7% | 49.9% |
| (16) | Any mental health service | 26.2% | 22.3% | 30.4% |
| (16) | Any general health service | 44.1% | 39.6% | 48.8% |
| (16) | Any health service | 54.6% | 49.9% | 59.3% |
| (16) | Any non-health service | 14.8% | 11.7% | 18.7% |
| (16) | Any service use | 58.7% | 53.9% | 63.3% |
| (17) | Any service use | 15.7% | 9.1% | 22.3% |
| (17) | Any service use | 31.4% | 24.2% | 38.6% |
| (17) | Any service use | 26.6% | 16.5% | 36.7% |
| (17) | Any service use | 29.7% | 17.9% | 41.5% |
| (17) | Any service use | 24.7% | 20.5% | 28.9% |
| (17) | Any service use | 37.8% | 24.1% | 51.5% |
| (17) | Any service use | 34.4% | 19.9% | 48.9% |
| (17) | Any service use | 37.6% | 15.2% | 60.0% |
| (17) | Any service use | 33.3% | 9.4% | 57.2% |
| (17) | Any service use | 36.0% | 27.5% | 44.5% |
| (18) | Any mental health service | 39.2% | 37.3% | 41.1% |
| (19) | Any mental health service | 8.2% | 5.6% | 10.8% |
| (20) | Any non-health service | 43.4% | 37.8% | 49.0% |
| (21) | Any health service | 30.1% | 20.8% | 39.4% |
| (21) | Any general health service | 8.6% | 2.9% | 14.3% |
| (21) | Any mental health service | 21.5% | 13.2% | 29.9% |
| (21) | Any health service | 36.3% | 29.7% | 42.9% |
| (21) | Any general health service | 14.2% | 9.4% | 19.0% |
| (21) | Any mental health service | 22.1% | 16.4% | 27.7% |
| (22) | Any non-health service | 35.2% | 25.4% | 45.0% |
| (23) | Any health service | 32.1% | 0.0% | 64.7% |
| (24) | Any health service | 55.1% | 47.1% | 63.1% |
| (24) | Any general health service | 18.4% | 12.1% | 24.6% |
| (24) | Any non-health service | 6.1% | 2.2% | 10.0% |
| (24) | Any mental health service | 14.3% | 8.6% | 19.9% |
| (25) | Any general health service | 51.2% | 44.4% | 58.0% |
| (25) | Any mental health service | 45.0% | 38.2% | 51.7% |
| (26) | Any health service | 61.7% | 55.1% | 68.3% |
| (27) | Any mental health service | 18.1% | 12.2% | 24.1% |
| (28) | Any mental health service | 40.5% | 34.4% | 46.6% |
| (28) | Any general health service | 40.9% | 34.8% | 47.0% |
| (28) | Any health service | 58.7% | 51.6% | 65.8% |
| (28) | Any service use | 59.1% | 52.0% | 66.2% |
| (28) | MAT - stringent | 13.4% | 9.1% | 17.6% |
| (28) | MAT - stringent | 8.9% | 5.4% | 12.5% |
| (28) | MAT - stringent | 15.0% | 10.5% | 19.4% |
| (29) | Any mental health service | 23.3% | 19.8% | 26.9% |
| (29) | Any general health service | 23.3% | 19.8% | 26.9% |
| (29) | Any non-health service | 6.1% | 4.1% | 8.1% |
| (29) | Any service use | 52.8% | 48.6% | 56.9% |
| (29) | Any general health service | 29.7% | 27.1% | 32.3% |
| (29) | Any non-health service | 8.3% | 6.8% | 9.9% |
| (29) | Any service use | 54.9% | 52.1% | 57.7% |
| (29) | Any mental health service | 42.0% | 39.2% | 44.8% |
| (29) | Any mental health service | 17.2% | 13.4% | 21.0% |
| (29) | Any mental health service | 17.4% | 13.7% | 21.1% |
| (29) | Any mental health service | 16.9% | 12.7% | 21.0% |
| (29) | Any mental health service | 13.0% | 6.2% | 19.9% |
| (29) | Any general health service | 21.0% | 16.8% | 25.1% |
| (29) | Any general health service | 34.8% | 30.2% | 39.4% |
| (29) | Any general health service | 36.9% | 31.6% | 42.3% |
| (29) | Any general health service | 17.4% | 9.6% | 25.1% |
| (29) | Any non-health service | 9.9% | 6.9% | 13.0% |
| (29) | Any non-health service | 2.5% | 0.8% | 4.3% |
| (29) | Any non-health service | 4.3% | 0.2% | 8.5% |
| (29) | Any service use | 48.1% | 43.0% | 53.2% |
| (29) | Any service use | 62.0% | 57.3% | 66.7% |
| (29) | Any service use | 59.6% | 54.1% | 65.0% |
| (29) | Any service use | 34.8% | 25.1% | 44.5% |
| (30) | Any mental health service | 50.8% | 45.4% | 56.2% |
| (31) | Any health service | 16.9% | 7.1% | 26.7% |
| (31) | Any general health service | 12.4% | 4.9% | 19.9% |
| (31) | Any mental health service | 4.0% | 0.0% | 8.5% |
| (31) | Any non-health service | 4.1% | 0.0% | 8.6% |
| (32) | Any mental health service | 39.8% | 31.7% | 47.9% |
| (33) | Any service use | 13.4% | 7.7% | 19.1% |
| (33) | Any health service | 10.6% | 5.1% | 16.1% |
| (33) | Any general health service | 5.1% | 2.4% | 7.8% |
| (33) | Any mental health service | 6.1% | 0.6% | 11.6% |
| (33) | Any non-health service | 7.1% | 1.6% | 12.6% |
| (34) | Any mental health service | 12.6% | 7.7% | 17.5% |
| (34) | Any general health service | 11.5% | 8.0% | 15.0% |
| (34) | Any health service | 22.1% | 16.4% | 27.8% |
| (34) | Any non-health service | 5.4% | 1.9% | 8.9% |
| (34) | Any service use | 26.1% | 19.6% | 32.6% |
| (35) | Any general health service | 19.0% | 3.9% | 34.1% |
| (35) | Any health service | 27.0% | 9.9% | 44.1% |
| (35) | Any mental health service | 35.0% | 16.7% | 53.3% |
| (36) | Any general health service | 70.5% | 64.2% | 76.8% |
| (36) | Any general health service | 54.7% | 47.4% | 62.1% |
| (37) | Any mental health service | 8.9% | 5.7% | 12.1% |
| (37) | Any general health service | 13.4% | 9.6% | 17.2% |
| (37) | Any non-health service | 16.8% | 12.6% | 21.0% |
| (37) | Any health service | 31.1% | 25.9% | 36.3% |
| (38) | Any service use | 56.3% | 34.4% | 78.1% |
| (38) | Any service use | 55.9% | 40.7% | 71.1% |
| (38) | Any service use | 52.5% | 37.3% | 67.8% |
| (38) | Any service use | 55.6% | 44.3% | 66.9% |
| (38) | Any mental health service | 22.0% | 12.0% | 31.9% |
| (38) | Any mental health service | 25.0% | 16.0% | 34.0% |
| (38) | Any mental health service | 23.0% | 14.7% | 31.9% |
| (38) | Any mental health service | 26.3% | 18.3% | 34.2% |
| (38) | Any general health service | 24.6% | 7.2% | 42.0% |
| (38) | Any general health service | 30.7% | 17.9% | 43.6% |
| (38) | Any general health service | 37.0% | 22.2% | 51.9% |
| (38) | Any general health service | 45.9% | 35.3% | 56.5% |
| (38) | Any non-health service | 23.9% | 0.0% | 48.3% |
| (38) | Any non-health service | 16.4% | 8.1% | 24.8% |
| (38) | Any non-health service | 9.5% | 1.9% | 17.1% |
| (38) | Any non-health service | 21.1% | 12.7% | 29.5% |
| (39) | Any service use | 58.0% | 49.0% | 67.0% |
| (40) | Any mental health service | 59.8% | 37.2% | 82.5% |
| (40) | Any mental health service | 68.8% | 45.0% | 92.6% |
| (40) | Any mental health service | 66.0% | 29.6% | 100.0% |
| (40) | Any mental health service | 68.7% | 38.2% | 99.2% |
| (40) | Any mental health service | 85.1% | 47.4% | 100.0% |
| (40) | Any mental health service | 39.7% | 0.0% | 82.7% |
| (41) | Any health service | 32.6% | 27.2% | 38.1% |
| (41) | Any general health service | 12.8% | 9.0% | 16.7% |
| (41) | Any mental health service | 17.2% | 11.8% | 22.6% |
| (41) | MAT - stringent | 17.7% | 13.3% | 22.1% |
| (42) | Any service use | 23.2% | 17.4% | 28.9% |
| (42) | Any service use | 22.7% | 17.5% | 28.0% |
| (43) | Any service use | 50.5% | 47.5% | 53.5% |
| (43) | Any service use | 65.5% | 63.6% | 67.4% |
| (44) | Any service use | 60.4% | 56.6% | 64.3% |
| (44) | Any general health service | 41.9% | 38.0% | 45.7% |
| (44) | Any mental health service | 25.4% | 10.9% | 39.8% |
| (45) | Any mental health service | 9.0% | 3.1% | 14.9% |
| (46) | Any service use | 22.7% | 14.2% | 31.2% |
| (46) | Any general health service | 18.0% | 10.2% | 25.8% |
| (46) | Any mental health service | 5.2% | 0.7% | 9.7% |
| (46) | Any non-health service | 12.4% | 5.7% | 19.1% |
| (47) | Any service use | 11.6% | 7.6% | 15.6% |
| (47) | Any health service use | 9.4% | 5.5% | 13.4% |
| (47) | Any general health service | 2.3% | 0.2% | 4.4% |
| (47) | Any mental health service | 4.7% | 2.2% | 7.7% |
| (47) | Any non-health service | 2.6% | 1.4% | 3.8% |
| (47) | MAT - stringent | 0.9% | 0.2% | 1.6% |
| (48) | Any mental health service | 14.3% | 7.1% | 21.5% |
| (49) | Any mental health service | 36.8% | 31.7% | 41.9% |
| (50) | Any health service | 24.5% | 20.4% | 28.6% |
| (51) | Any mental health service | 28.9% | 7.4% | 50.5% |
| (51) | Any service use | 44.0% | 37.9% | 50.1% |
| (52) | Any health service use | 27.0% | 19.9% | 32.8% |
| (53) | Any mental health service | 22.1% | 17.2% | 27.0% |
| (53) | Any mental health service | 14.8% | 9.4% | 20.1% |
| (53) | Any mental health service | 19.0% | 10.0% | 27.9% |
| (53) | Any mental health service | 29.4% | 20.6% | 38.2% |
| (53) | Any mental health service | 22.6% | 14.2% | 31.1% |
| (53) | Any mental health service | 37.4% | 33.1% | 41.7% |
| (53) | Any mental health service | 32.0% | 24.5% | 39.5% |
| (53) | Any mental health service | 21.4% | 15.1% | 27.7% |
| (53) | Any mental health service | 26.0% | 16.8% | 35.2% |
| (53) | Any mental health service | 27.8% | 24.0% | 31.6% |
| (53) | Any mental health service | 27.1% | 14.3% | 39.9% |
| (53) | Any mental health service | 51.5% | 42.6% | 60.4% |
| (53) | Any mental health service | 47.9% | 40.4% | 55.3% |
| (53) | Any mental health service | 47.6% | 38.1% | 57.1% |
| (53) | Any mental health service | 40.0% | 35.7% | 44.3% |
| (53) | Any mental health service | 39.6% | 31.5% | 47.6% |
| (53) | Any mental health service | 40.1% | 25.5% | 54.8% |
| (53) | Any mental health service | 65.8% | 56.0% | 75.6% |
| (53) | Any mental health service | 51.1% | 42.0% | 60.2% |
| (53) | Any mental health service | 54.1% | 49.4% | 58.8% |
| (53) | Any mental health service | 8.7% | 5.6% | 11.8% |
| (53) | Any mental health service | 60.1% | 54.0% | 66.2% |
| (53) | Any mental health service | 58.4% | 54.6% | 62.3% |
| (53) | MAT - lenient | 15.2% | 10.9% | 19.4% |
| (53) | MAT - lenient | 8.4% | 4.2% | 12.6% |
| (53) | MAT - lenient | 2.1% | 0.0% | 5.4% |
| (53) | MAT - lenient | 16.1% | 9.0% | 23.2% |
| (53) | MAT - lenient | 13.3% | 6.4% | 20.1% |
| (53) | MAT - lenient | 30.1% | 26.0% | 34.1% |
| (53) | MAT - lenient | 17.3% | 11.2% | 23.4% |
| (53) | MAT - lenient | 15.8% | 10.3% | 21.4% |
| (53) | MAT - lenient | 20.5% | 12.0% | 28.9% |
| (53) | MAT - lenient | 15.2% | 10.6% | 19.7% |
| (53) | MAT - lenient | 19.4% | 7.9% | 30.8% |
| (53) | MAT - lenient | 40.8% | 32.0% | 49.6% |
| (53) | MAT - lenient | 40.3% | 32.9% | 47.6% |
| (53) | MAT - lenient | 43.0% | 33.6% | 52.4% |
| (53) | MAT - lenient | 30.2% | 26.2% | 34.3% |
| (53) | MAT - lenient | 34.4% | 26.6% | 42.3% |
| (53) | MAT - lenient | 35.8% | 21.5% | 50.1% |
| (53) | MAT - lenient | 51.0% | 40.6% | 61.3% |
| (53) | MAT - lenient | 46.8% | 37.7% | 55.8% |
| (53) | MAT - lenient | 42.4% | 37.8% | 47.1% |
| (53) | MAT - lenient | 48.4% | 42.9% | 53.9% |
| (53) | MAT - lenient | 53.0% | 46.8% | 59.2% |
| (53) | MAT - lenient | 47.4% | 43.5% | 51.4% |
| (54) | MAT - stringent | 28.4% | 25.9% | 30.9% |
| (54) | MAT - lenient | 37.3% | 34.0% | 40.5% |
| (54) | MAT - moderate | 24.9% | 21.8% | 28.1% |
| (54) | MAT - stringent | 29.1% | 26.6% | 31.6% |
| (30) | Any mental health service | 21.3% | 18.1% | 24.4% |
| (55) | Any mental health service | 44.0% | 37.9% | 50.1% |
| (56) | Any mental health service | 31.6% | 27.7% | 35.5% |
| (56) | Any general health service | 27.2% | 22.4% | 32.0% |
| (56) | Any health service | 54.6% | 46.1% | 57.2% |
| (56) | Any non-health service | 12.2% | 6.2% | 18.2% |
| (56) | Any service use | 57.3% | 51.9% | 62.8% |
| (56) | MAT - stringent | 21.7% | 18.1% | 25.2% |
| (57) | Any mental health service | 19.9% | 14.9% | 24.9% |
| (57) | Any general health service | 73.4% | 67.8% | 79.0% |
| (57) | Any health service | 11.6% | 7.6% | 15.6% |
| (57) | Any non-health service | 20.2% | 15.1% | 25.3% |
| (23) | MAT - lenient | 35.8% | 25.0% | 46.5% |
| (23) | Any mental health service | 34.4% | 28.7% | 40.2% |
| (23) | Any general health service | 30.0% | 24.4% | 35.5% |
| (58) | Any mental health service | 24.8% | 3.1% | 46.4% |
| (59) | Any mental health service | 28.8% | 24.5% | 33.1% |
| (60). | Any mental health service | 0.0% | 0.0% | 0.1% |
| (61) | Any mental health service | 29.8% | 22.5% | 37.0% |
| (61) | Any health service | 35.7% | 25.6% | 45.7% |
| (61) | Any non-health service | 4.9% | 1.6% | 8.1% |
| (61) | Any service use | 38.5% | 28.7% | 48.3% |
| (62) | Any mental health service | 11.1% | 0.0% | 31.6% |
| (62) | Any health service | 66.7% | 35.9% | 97.5% |
| (63) | Any non-health service | 45.3% | 36.3% | 54.3% |
| (63) | Any mental health service | 50.9% | 43.3% | 58.4% |
| (64) | Any mental health service | 56.1% | 23.7% | 88.5% |
| (65) | Any health service | 77.0% | 70.1% | 84.0% |
| (65) | Any mental health service | 61.9% | 54.4% | 69.3% |
| (65) | Any service use | 79.6% | 73.5% | 85.7% |
| (66) | Any mental health service | 18.2% | 13.2% | 23.2% |
| (67) | Any mental health service | 36.0% | 35.3% | 36.7% |
| (67) | Any mental health service | 34.9% | 34.2% | 35.6% |
| (67) | Any mental health service | 34.2% | 33.5% | 34.9% |
| (67) | Any mental health service | 38.6% | 37.9% | 39.3% |
| (67) | Any mental health service | 36.0% | 35.3% | 36.7% |
| (67) | Any mental health service | 39.2% | 38.5% | 39.9% |
| (67) | Any mental health service | 15.3% | 14.8% | 15.8% |
| (67) | Any mental health service | 15.8% | 15.3% | 16.3% |
| (67) | Any mental health service | 16.9% | 16.4% | 17.4% |
| (67) | Any mental health service | 20.0% | 19.4% | 20.6% |
| (67) | Any mental health service | 18.2% | 17.7% | 18.7% |
| (67) | Any mental health service | 21.3% | 20.7% | 21.9% |
| (68) | Any mental health service | 18.3% | 12.6% | 24.0% |
| (68) | Any mental health service | 11.1% | 8.2% | 14.0% |
| (69) | Any health service | 20.8% | 15.3% | 27.5% |
| (70) | Any mental health service | 27.9% | 7.7% | 48.2% |
| (70) | Any mental health service | 27.7% | 7.8% | 47.5% |
| (70) | Any non-health service | 20.2% | 11.7% | 28.7% |
| (70) | Any non-health service | 16.0% | 9.9% | 22.2% |
| (70) | Any service use | 51.2% | 37.6% | 64.7% |
| (70) | Any health service | 25.3% | 15.0% | 39.4% |
| (70) | Any general health service | 15.6% | 7.9% | 28.3% |
| (70) | Any service use | 62.6% | 51.6% | 72.4% |
| (70) | Any health service | 14.3% | 8.9% | 22.1% |
| (70) | Any general health service | 25.6% | 18.1% | 34.9% |
| (71) | Any service use | 38.2% | 36.3% | 40.2% |
| (72) | Any general health service | 52.3% | 46.0% | 58.6% |
| (72) | Any mental health service | 39.8% | 33.6% | 46.0% |
| (73) | Any health service | 87.7% | 83.5% | 91.9% |
| (73) | Any general health service | 80.7% | 75.7% | 85.7% |
| (73) | Any mental health service | 70.5% | 64.7% | 76.3% |
| (74) | Any non-health service | 55.1% | 46.2% | 64.1% |
| (74) | Any mental health service | 60.5% | 9.0% | 100.0% |
| (75) | Any health service | 50.2% | 38.8% | 61.4% |
| (76) | Any health service | 54.0% | 24.0% | 83.6% |
| (77) | Any health service | 3.7% | 2.0% | 5.4% |
| (78) | Any health service | 52.7% | 35.7% | 69.4% |
| (79) | Any health service | 49.6% | 33.2% | 65.7% |
| (80) | Any health service | 36.8% | 30.7% | 42.8% |
| (81) | Any health service | 11.9% | 7.0% | 16.7% |
| (82) | Any health service | 8.6% | 5.1% | 12.0% |
| (83) | Any health service | 1.5% | 0.0% | 4.7% |
| (84) | Any health service | 11.8% | 4.1% | 19.3% |
| (85) | Any health service | 12.3% | 4.8% | 19.6% |
| (86) | Any health service | 63.5% | 44.6% | 82.0% |
| (87) | Any health service | 15.1% | 2.5% | 27.5% |
| (88) | Any health service | 75.3% | 49.5% | 100.0% |
| (89) | Any health service | 5.7% | 2.6% | 8.7% |
| (90) | Any health service | 23.1% | 15.8% | 30.4% |
| (91) | Any health service | 20.6% | 8.3% | 32.8% |
| (92) | Any health service | 57.8% | 42.4% | 73.0% |
| (93) | Any health service | 19.0% | 11.9% | 26.0% |
| (94) | Any health service | 77.3% | 51.9% | 100.0% |
| (95) | Any health service | 4.0% | 1.2% | 6.7% |
| (96) | Any health service | 15.5% | 5.2% | 25.7% |
| (97) | Any health service | 6.6% | 4.5% | 8.7% |
| (98) | Any health service | 60.2% | 35.1% | 84.9% |
| (99) | Any health service | 56.2% | 36.6% | 75.5% |
| (100) | Any health service | 7.8% | 2.6% | 13.1% |
| (101) | Any health service | 6.1% | 2.8% | 9.4% |
| (102) | Any health service | 33.4% | 17.9% | 48.7% |
| (103) | Any health service | 45.9% | 26.9% | 64.6% |
| (104) | Any health service | 49.3% | 27.2% | 71.0% |
| (105) | Any health service | 0.0% | 0.0% | 0.1% |
| (106) | Any health service | 12.6% | 4.9% | 20.3% |
| (107) | Any health service | 6.7% | 1.6% | 11.8% |
| (108) | Any health service | 35.4% | 24.6% | 46.0% |
| (109) | Any health service | 31.9% | 24.7% | 39.0% |
| (110) | Any health service | 7.9% | 5.6% | 10.2% |
| (111) | Any health service | 10.4% | 0.0% | 21.1% |
| (112) | Any health service | 28.1% | 19.0% | 37.1% |
| (113) | Any health service | 37.1% | 23.5% | 50.5% |
| (114) | Any health service | 30.5% | 12.4% | 48.4% |
| (115) | Any health service | 14.0% | 10.0% | 17.9% |
| (116) | Any health service | 17.0% | 11.3% | 22.6% |
| (117) | Any health service | 12.4% | 7.7% | 17.0% |
| (118) | Any health service | 76.0% | 62.4% | 89.4% |
| (119) | Any health service | 39.9% | 31.6% | 48.2% |
| (120) | Any health service | 14.7% | 9.3% | 20.1% |
| (121) | Any health service | 9.9% | 2.6% | 17.1% |
| (122) | Any health service | 8.4% | 0.0% | 16.8% |
| (123) | Any health service | 26.6% | 7.2% | 45.7% |
| (124) | Any health service | 21.4% | 9.9% | 32.7% |
| (125) | Any health service | 65.0% | 55.6% | 74.2% |
| (126) | Any health service | 9.8% | 3.5% | 16.0% |
| (127) | Any health service | 46.0% | 35.3% | 56.5% |
| (128) | Any health service | 32.8% | 18.7% | 46.6% |
| (129) | Any health service | 15.1% | 10.5% | 19.5% |
| (130) | Any health service | 13.5% | 7.2% | 19.8% |
| (131) | Any health service | 4.4% | 0.0% | 10.3% |
| (132) | Any health service | 58.4% | 42.3% | 74.2% |
| (133) | Any health service | 35.9% | 23.5% | 48.0% |
| (134) | Any health service | 17.9% | 0.0% | 40.7% |
| (135) | Any health service | 7.3% | 2.9% | 11.6% |
| (136) | Any health service | 7.6% | 1.5% | 13.7% |

Note: Multiple estimates by treatment type and study may represent sex and/or age-specific estimates. MAT = Minimally adequate treatment.

1. **Study characteristics:**

Treatment rates for dysthymia, depressive disorders combined, and mood disorders are reported separately. The level of service use investigated varied considerably between studies. Only a few countries (largely high-income countries) had treatment rates available for what we considered to be the most informative group i.e. aggregated treatment rates for any health service as well as either mental health service or minimally adequate treatment. The majority of countries had aggregated treatment rates available for any health service only, with large parts of the world with no data available. Treatment rates for non-health based services were generally reported in combination with rates of any service use, except for Singapore where one study focused non-health based services for MDD.

MDD Treatment Rates:

Any service use: There were 44 data-points pertaining to overall treatment rates of any service, from which we estimated treatment gaps for MDD, irrespective of whether the service was in the health or non-health sector. Treatment rates for the utilization of any service ranged from 79.6% [73.5, 85.7] in Australia to 11.6% [7.6, 15.6] in China.

Any health service use: Treatment rates for any health service use were lowest in Malawi, China, Bangladesh, Ghana, and the UAE, and the highest in Denmark, Portugal, Australia, Germany, and Latvia. Treatment rates for any health service utilization ranged from 87.7% [83.5, 91.8] in Latvia to 0% (indicating no treatment) in Malawi. Fig 4 presents treatment gaps for MDD estimated for any heath service utilization and location. Although there were many parts of the world with missing data, we found a statistically significant effect by income group in our analyses. Treatment rates were significantly lower in middle and low income countries compared to high income countries.

Any general health service use: There were 37 data-points available on treatment rates of general health services, (i.e., services provided by general practitioners or other medical doctor). Treatment rates for any general health service use ranged from 80.7% [75.7, 85.7] in Latvia to 2.3% [<0.1, 4.4] in China.

Any mental health service use: Treatment rates for any mental health service utilization ranged from 85.1% [47.4, 100.0] in Australia to 0% (Indicating no treatment) in Laos PDR for MDD. Pooled estimates reported in the paper by World Bank income level showed significant differences in treatment gaps between income groups.

Any non-health service use: Treatment rates for non-health services tended to be low. These ranged from 55.1% [46.2, 64.1] in Australia to 2.5 % [0.08, 4.3] in Canada for MDD.

Minimally adequate treatment: There were 39 data-points available on rates of minimally adequate treatment. The majority of estimates also came from high-income countries (28 data points from high-income countries; 10 data points from middle-income countries; 1 data point from low income countries). The criteria used to define minimally adequate treatment varied considerably between studies and therefore these data were also analyzed separately. The key sources of variation included: (1) Whether or not a visit duration was specified; and (2) How ‘psychotherapy’ or ‘psychological treatment’ was operationalized, either according to the type of provider seen or therapy received. Based on the available definitions reported by studies that reported on MAT, we pooled rates for MAT definitions that were either considered stringent or non-stringent.

1. **Meta-regression Coefficients (By Super-region):**

**Table S5. Regression Coefficients and 95% uncertainty intervals for MDD treatment rates modeled as a function of select covariates (treatment type, age, sex, location, and survey instrument)**

| **Covariate** | **Parameter Estimate [95% UI]** | **P-Value** |
| --- | --- | --- |
| Intercept ^a^ | 0.62 [0.60, 0.64] | <0.001 |
| **Treatment Type^a^** | |  |
| Any service use | 0.28 [0.27, 0.29] | <0.001 |
| General health service use | 0.06 [0.05, 0.08] | <0.001 |
| Minimally Adequate Treatment (MAT) | -0.12 [-0.13, -0.10] | <0.001 |
| Any health service use | 0.17 [0.16, 0.19] | <0.001 |
| Any non-health service use | -0.16 [-0.17, -0.14] | <0.001 |
| **Sample characteristics** | |  |
| Age | 0.001 [< 0.001, 0.0016] | <0.001 |
| Percent Female | 0.06 [0.04, 0.07] | <0.001 |
| **GBD Super Region^a^** | |  |
| Central Europe, Eastern Europe & Central Asia | -0.06 [-0.13, 0.02] | 0.148 |
| Latin America and Caribbean | -0.15 [-0.25, -0.06] | 0.002 |
| North Africa and the Middle East, and Sub-Saharan Africa^b^ | -0.33 [-0.41, -0.25] | <0.001 |
| South Asia, and Southeast Asia, East Asia, and Oceania^b^ | -0.31 [-0.4, -0.22] | <0.001 |
| **Methodological Covariates** | |  |
| Survey instrument | -0.10 [-0.17, -0.04] | 0.001 |

Note: 95% UIs incorporate between-study heterogeneity ^a^ Intercept represents mental health service use in High Income locations. ^b^ Represents two GBD super-regions combined due to limited data from each super-region.

1. **Supplementary Results:**

Treatment rates disaggregated by MDD severity vary for those with mild, moderate, or severe MDD and were therefore analyzed separately as part of the supplementary analyses. Minimally adequate treatment rates were also expected to vary by scope of MAT definitions. Therefore, analyses with MAT definitions were also conducted as part of our supplementary analyses (see below).

Dysthymia, depressive disorders, and mood disorders were expected to have different prevalence and treatment profiles and were therefore reported separately in our supplementary analyses (see appendix). For MDD data, initial model iterations were tested with year as a covariate to assess changes in treatment rates over time. However, there were very few countries (particularly in the low income group) with data spanning enough time points. Therefore, we chose to examine a few select countries (USA, Canada, Netherlands, and Australia) with relatively more data points by year to examine trends in treatment rates over time. For years with missing data, a spline interpolation with low degree polynomials was used to impute treatment coverage in order to better assess trends over a complete time series.

7.1 Treatment by Disorder severity: There were 55 datapoints for MDD from high-income countries. There were 23 estimates available for severe disorders, and 16 estimates for disorders moderate and mild severity respectively. Treatment rates were higher for all treatment types for severe MDD compared to moderate and mild MDD (See Fig S1).

7.2 Treatment by Year: We examined treatment rates by year for the United States, Canada, Netherlands, and Australia between 2002 and 2016. Based on data from the United States, estimated treatment rates increased between 34.6% [30.4, 38.5] in 2002 to 63.8% in 2007 [60.8, 66.8] Estimated treatment rates by year for the remaining countries are in Fig S2.

7.3 Treatment rates by MAT definition type: There were 39 datapoints for minimally adequate treatment for MDD. There were 13 datapoints with stringent definitions of MAT and 26 datapoints with non-stringent definitions of MAT. We examined variation in MAT rates by income group and definition stringency. The small number of data points precluded further analysis by other covariates. Overall, MAT rates were higher in high income locations compared to middle and low-income locations and MAT rates for stringent care thresholds were lower compared to those for non-stringent thresholds. In high income locations, the pooled MAT rate was 31.6% [9.7%, 56.0%] for stringent thresholds and 34.5% [12.2%, 59.0%] for non-stringent thresholds. In low and middle locations, the pooled MAT rate was 0.8% [<0.1%, 10.7%] for stringent thresholds and 15.4% [4.6%, 29.6%] for non-stringent thresholds.

7.4 Treatment rates for Dysthymia, Depressive Disorders, and Mood Disorders: Dysthymia Treatment Rates: There were 11 studies (31 observations) reporting treatment rates for dysthymia. Data were from 9 high income and 2 from upper-middle income countries. Pooled treatment rates for dysthymia were higher for high-income countries 36.5% [29.5, 43.5] compared to upper-middle income countries 25.7% [17.4%, 34.1%].

Depressive Disorders Treatment Rates: There were 12 studies (44 observations) reporting treatment rates for depressive disorders as an aggregated group. Data from high-income countries originated from Western Europe and the United States. Upper-middle income countries included China, Mexico, Russia, and South Africa. Lower-middle income countries included Ghana and India. Differences in treatment rates by income group were similar to those for MDD and dysthymia. Pooled treatment rates for depressive disorders were significantly higher for high-income locations at 17.5% [5.7%, 33.4%] compared to upper-middle income at 18.5% [7.3%, 32.7%] and lower-middle income locations at 13.1% [3.8%, 26.5%].

Mood Disorders Treatment Rates: There were 36 studies (126 observations) reporting treatment rates for mood disorders. High income countries in this dataset included Australia, Belgium, Czech Republic, Israel, Japan, New Zealand, Spain, Saudi Arabia, and the United States. Upper-middle income countries in this dataset included China, Argentina, Brazil, Colombia, Guatemala, Lebanon, Mexico, Peru, and South Africa. There were twenty four studies that used data from the World Mental Health surveys. Sensitivity analyses did not reveal any significant differences between treatment rates from WMHS compared to other studies when controlling for income group (p-value = 0.410). The pooled treatment rate for mood disorders were 31.5% [9.9%, 57.5%] for high income countries and 15.1% [1.3%, 39.0%] for upper-middle income countries.

- 1. Funnel Plots: Risk of publication bias was assessed using funnel plots (See Fig S3 and Fig S4).


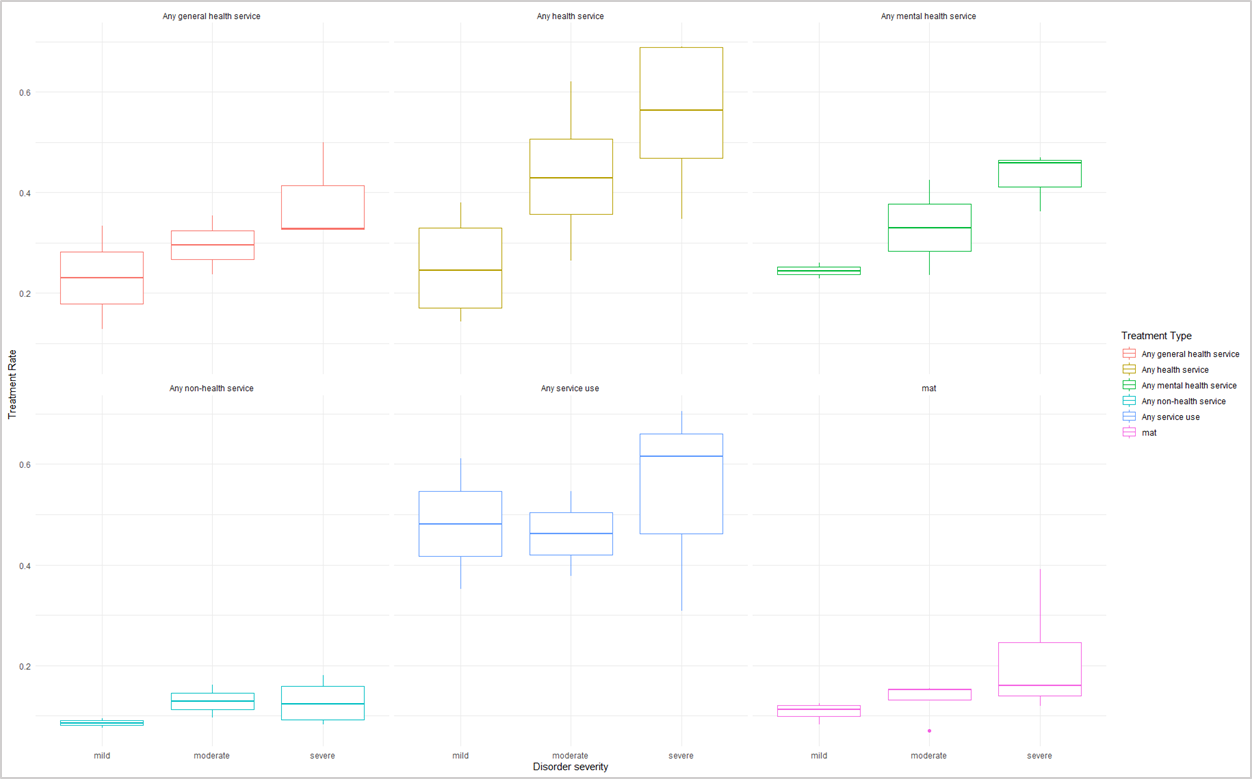


Note: MAT: Minimally adequate treatment; Notch represents the median and 95% confidence interval around the median (+/- 1.58 the interquartile range / sqrt(n)). Lower and upper hinges correspond to the 25^th^ and 75^th^ percentiles respectively.

Fig S1. Treatment rates by MDD severity and treatment type


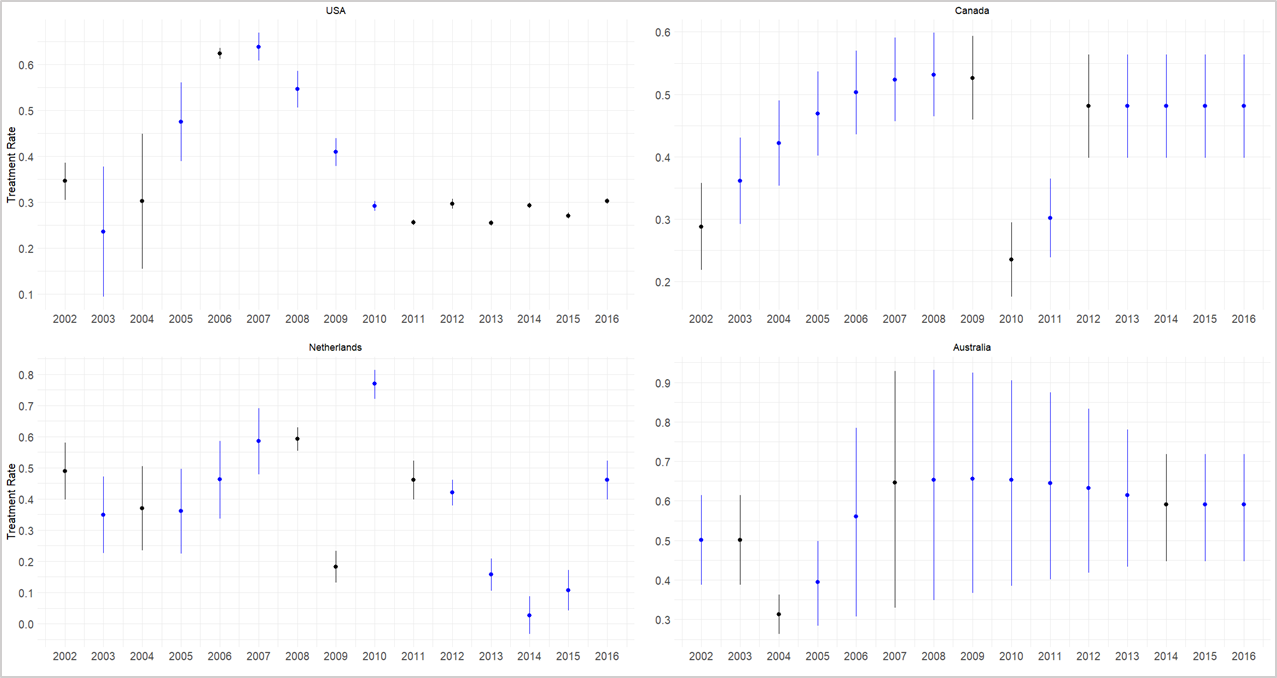


Note: Blue = interpolated; Black = Observed.

Fig S2: MDD Treatment rates by year; USA, Canada, Netherlands, Australia; 2002-2016


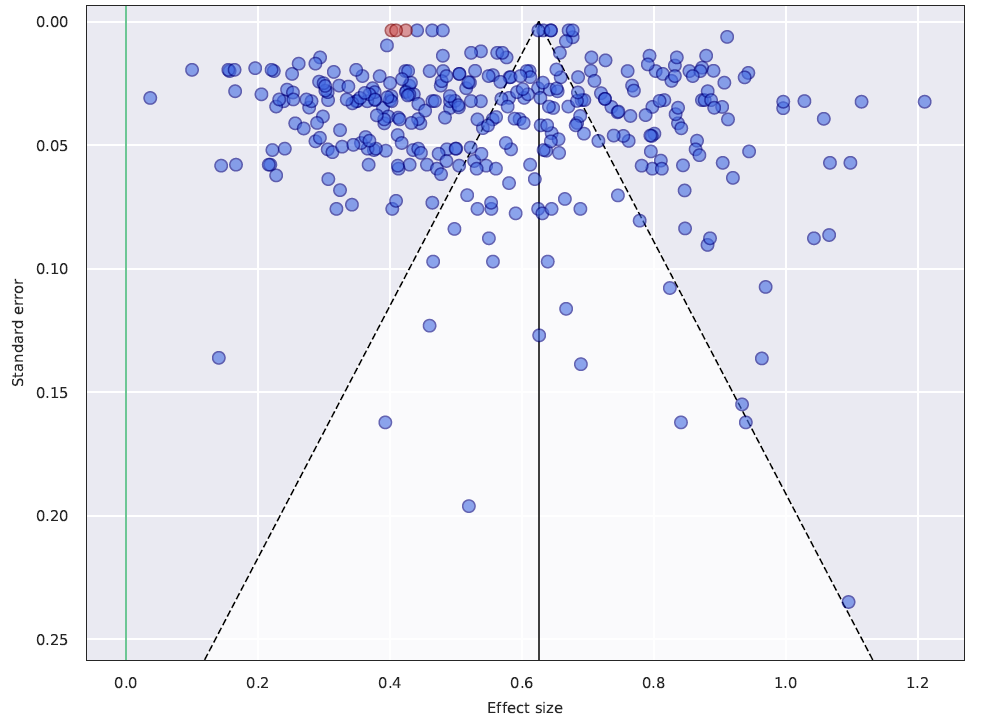


Note. Red datapoints represent outliers detected by meta-regression.

Fig S3: Funnel plot for meta-regression of treatment rates for major depressive disorder by income status


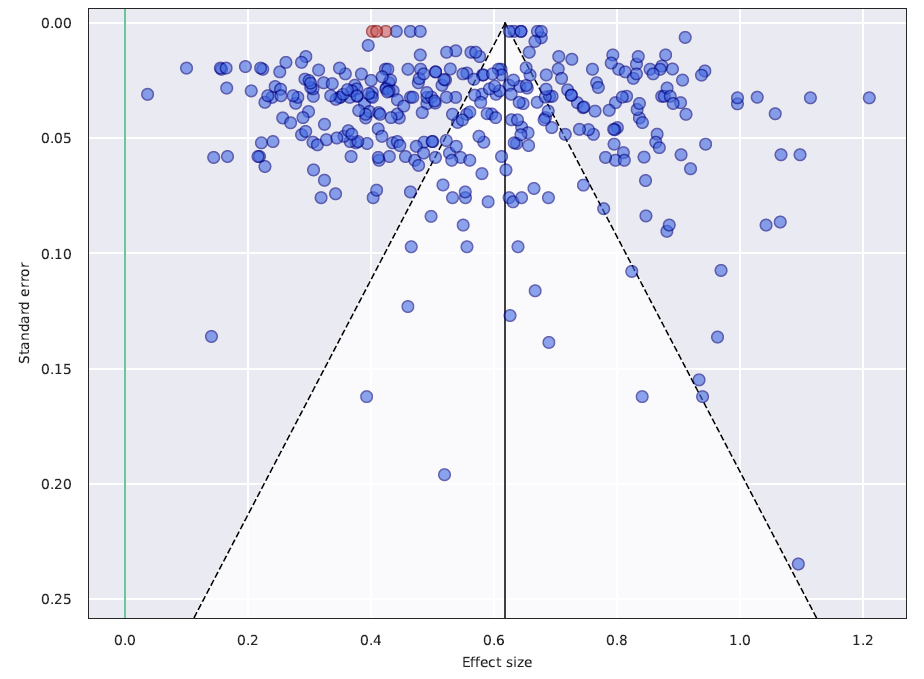


Note. Red datapoints represent outliers detected by meta-regression.

Fig S4: Funnel plot for meta-regression of treatment rates for major depressive disorder by GBD super-region

References:

1. GBD 2015 DALYs and HALE Collaborators. Global, regional, and national disability-adjusted life-years (DALYs) for 315 diseases and injuries and healthy life expectancy (HALE), 1990–2015: a systematic analysis for the Global Burden of Disease Study 2015. Lancet. 2016;388:1603-58.

2. GBD 2015 Disease and Injury Incidence and Prevalence Collaborators. Global, regional, and national incidence, prevalence, and years lived with disability for 310 diseases and injuries, 1990–2015: a systematic analysis for the Global Burden of Disease Study 2015. Lancet. 2016;388:1545-602.

3. Patel V, Chisholm D, Parikh R, Charlson FJ, Degenhardt L, Dua T, et al. Addressing the burden of mental, neurological, and substance use disorders: key messages from Disease Control Priorities, 3rd edition. Lancet. 2016;387(10028):1672-85.

4. Lancet Global Mental Health G, Chisholm D, Flisher AJ, Lund C, Patel V, Saxena S, et al. Scale up services for mental disorders: a call for action. Lancet. 2007;370(9594):1241-52.

5. Patel V, Boyce N, Collins PY, Saxena S, Horton R. A renewed agenda for global mental health. Lancet. 2011;378(9801):1441-2.

6. Saxena S, Funk M, Chisholm D. World Health Assembly adopts Comprehensive Mental Health Action Plan 2013-2020. Lancet. 2013;381(9882):1970-1.

7. Izutsu T, Tsutsumi A, Minas H, Thornicroft G, Patel V, Ito A. Mental health and wellbeing in the Sustainable Development Goals. Lancet Psychiatry. 2015;2(12):1052-4.

8. Organization WH. Mental Health and Substance Use. Health in 2015: from MDGs, Millennium Development Goals to SDGs, Sustainable Development Goals. Switzerland: World Health Organization; 2015.

9. Kohn R, Saxena S, Levav I, Saraceno B. The treatment gap in mental health care. Bull World Health Organ. 2004;82(11):858-66.

10. Wang PS, Lane M, Olfson M, Pincus HA, Wells KB, Kessler RC. Twelve-month use of mental health services in the United States: results from the National Comorbidity Survey Replication. Arch Gen Psychiatry. 2005;62(6):629-40.

11. ten Have M, de Graaf R, van Dorsselaer S, Beekman A. Lifetime treatment contact and delay in treatment seeking after first onset of a mental disorder. Psychiatr Serv. 2013;64(10):981-9.

12. Kleinberg A, Aluoja A, Vasar V. Help-seeking for emotional problems in major depression : findings of the 2006 Estonian health survey. Community Ment Health J. 2013;49(4):427-32.

13. Levinson D, Lerner Y, Zilber N, Grinshpoon A, Levav I. Twelve-month service utilization rates for mental health reasons: data from the Israel National Health Survey. Isr J Psychiatry Relat Sci. 2007;44(2):114-25.

14. Mojtabai R. Unmet need for treatment of major depression in the United States. Psychiatr Serv. 2009;60(3):297-305.

15. Naganuma Y, Tachimori H, Kawakami N, Takeshima T, Ono Y, Uda H, et al. Twelve-month use of mental health services in four areas in Japan: findings from the World Mental Health Japan Survey 2002-2003. Psychiatry Clin Neurosci. 2006;60(2):240-8.

16. Oakley Browne MA, Wells JE, McGee MA. Twelve-month and lifetime health service use in Te Rau Hinengaro: The New Zealand Mental Health Survey. Aust N Z J Psychiatry. 2006;40(10):855-64.

17. Rafful C, Medina-Mora ME, Borges G, Benjet C, Orozco R. Depression, gender, and the treatment gap in Mexico. J Affect Disord. 2012;138(1-2):165-9.

18. Kokaua J, Wells JE. Twelve-month prevalences of mental disorders and treatment contact among Cook Islanders resident in New Zealand. Pacific health dialog. 2009;15(1):79-88.

19. Tomlinson M, Grimsrud AT, Stein DJ, Williams DR, Myer L. The epidemiology of major depression in South Africa: results from the South African stress and health study. S Afr Med J. 2009;99(5 Pt 2):367-73.

20. Wahlstrom M, Sihvo S, Haukkala A, Kiviruusu O, Pirkola S, Isometsa E. Use of mental health services and complementary and alternative medicine in persons with common mental disorders. Acta psychiatrica Scandinavica. 2008;118(1):73-80.

21. Hamalainen J, Isometsa E, Sihvo S, Pirkola S, Kiviruusu O. Use of health services for major depressive and anxiety disorders in Finland. Depression and anxiety. 2008;25(1):27-37.

22. Feng L, Chiam PC, Kua EH, Ng TP. Use of complementary and alternative medicines and mental disorders in community-living Asian older adults. Archives of gerontology and geriatrics. 2010;50(3):243-9.

23. Fernández A, Haro JM, Codony M, Vilagut G, Martínez-Alonso M, Autonell J, et al. Treatment adequacy of anxiety and depressive disorders: primary versus specialised care in Spain. J Affect Disord. 2006;96(1-2):9-20.

24. Fleury MJ, Grenier G, Bamvita JM, Caron J. Determinants and patterns of service utilization and recourse to professionals for mental health reasons. BMC Health Serv Res. 2014;14:161.

25. Fleury MJ, Grenier G, Bamvita JM, Perreault M, Caron J. Determinants associated with the utilization of primary and specialized mental health services. Psychiatr Q. 2012;83(1):41-51.

26. Fleury MJ, Grenier G, Bamvita JM, Perreault M, Kestens Y, Caron J. Comprehensive determinants of health service utilisation for mental health reasons in a Canadian catchment area. Int J Equity Health. 2012;11:20.

27. Garrido MM, Kane RL, Kaas M, Kane RA. Use of mental health care by community-dwelling older adults. Journal of the American Geriatrics Society. 2011;59(1):50-6.

28. Gabilondo A, Rojas-Farreras S, Rodríguez A, Fernández A, Pinto-Meza A, Vilagut G, et al. Use of primary and specialized mental health care for a major depressive episode in Spain by ESEMeD respondents. Psychiatr Serv. 2011;62(2):152-61.

29. Gadalla TM. Comparison of users and non-users of mental health services among depressed women: a national study. Women & health. 2008;47(1):1-19.

30. Gonzalez HM, Vega WA, Williams DR, Tarraf W, West BT, Neighbors HW. Depression care in the United States: too little for too few. Arch Gen Psychiatry. 2010;67(1):37-46.

31. Gureje O, Uwakwe R, Oladeji B, Makanjuola VO, Esan O. Depression in adult Nigerians: results from the Nigerian Survey of Mental Health and Well-being. J Affect Disord. 2010;120(1-3):158-64.

32. Gwynn RC, McQuistion HL, McVeigh KH, Garg RK, Frieden TR, Thorpe LE. Prevalence, diagnosis, and treatment of depression and generalized anxiety disorder in a diverse urban community. Psychiatric Services (Washington, DC). 2008;59(6):641-7.

33. Al-Habeeb A, Altwaijri YA, Al-Subaie AS, Bilal L, Almeharish A, Sampson NA, et al. Twelve-month treatment of mental disorders in the Saudi National Mental Health Survey. Int J Methods Psychiatr Res. 2020;29(3):e1832.

34. Borges G, Medina-Mora ME, Wang PS, Lara C, Berglund P, Walters E. Treatment and adequacy of treatment of mental disorders among respondents to the Mexico National Comorbidity Survey. American journal of psychiatry. 2006;163(8):1371-8.

35. Bogner HR, de Vries HF, Maulik PK, Unutzer J. Mental health services use: Baltimore epidemiologic catchment area follow-up. The American journal of geriatric psychiatry : official journal of the American Association for Geriatric Psychiatry. 2009;17(8):706-15.

36. Brugha TS, Bebbington PE, Singleton N, Melzer D, Jenkins R, Lewis G, et al. Trends in service use and treatment for mental disorders in adults throughout Great Britain. Br J Psychiatry. 2004;185:378-84.

37. Cole MG, McCusker J, Sewitch M, Ciampi A, Dyachenko A. Health services use for mental health problems by community-living seniors with depression. International psychogeriatrics / IPA. 2008;20(3):554-70.

38. Cheung AH, Dewa CS. Mental health service use among adolescents and young adults with major depressive disorder and suicidality. Canadian journal of psychiatry Revue canadienne de psychiatrie. 2007;52(4):228-32.

39. Byers AL, Arean PA, Yaffe K. Low use of mental health services among older Americans with mood and anxiety disorders. Psychiatric services (Washington, DC). 2012;63(1):66-72.

40. Hollingworth SA, Burgess PM, Whiteford HA. Affective and anxiety disorders: prevalence, treatment and antidepressant medication use. The Australian and New Zealand journal of psychiatry. 2010;44(6):513-9.

41. Hamalainen J, Isometsa E, Sihvo S, Kiviruusu O, Pirkola S, Lonnqvist J. Treatment of major depressive disorder in the Finnish general population. Depress Anxiety. 2009;26(11):1049-59.

42. Hailemariam S, Tessema F, Asefa M, Tadesse H, Tenkolu G. The prevalence of depression and associated factors in Ethiopia: findings from the National Health Survey. Int J Ment Health Syst. 2012;6(1):23.

43. Hasin DS, Goodwin RD, Stinson FS, Grant BF. Epidemiology of major depressive disorder: results from the National Epidemiologic Survey on Alcoholism and Related Conditions. Arch Gen Psychiatry. 2005;62(10):1097-106.

44. Kovess-Masfety V, Briffault X, Sapinho D. Prevalence, risk factors, and use of health care in depression: a survey in a large region of France between 1991 and 2005. Canadian journal of psychiatry Revue canadienne de psychiatrie. 2009;54(10):701-9.

45. Mugisha J, Muyinda H, Malamba S, Kinyanda E. Major depressive disorder seven years after the conflict in northern Uganda: burden, risk factors and impact on outcomes (The Wayo-Nero Study). BMC Psychiatry. 2015;15(48).

46. Lee S, Tsang A, Huang YQ, He YL, Liu ZR, Zhang MY, et al. The epidemiology of depression in metropolitan China. Psychol Med. 2009;39(5):735-47.

47. Lu J, Xu X, Huang Y, Li T, Ma C, Xu G, et al. Prevalence of depressive disorders and treatment in China: a cross-sectional epidemiological study. Lancet Psychiatry. 2021;8(11):981-90.

48. Nisar N, Billoo N, Gadit AA. Prevalence of depression and the associated risks factors among adult women in a fishing community. J Pak Med Assoc. 2004;54(10):519-25.

49. Honkonen T, Virtanen M, Ahola K, Kivimäki M, Pirkola S, Isometsä E, et al. Employment status, mental disorders and service use in the working age population. Scand J Work Environ Health. 2007;33(1):29-36.

50. Lee S, Tsang A, Kwok K. Twelve-month prevalence, correlates, and treatment preference of adults with DSM-IV major depressive episode in Hong Kong. J Affect Disord. 2007;98(1-2):129-36.

51. Singleton N, Bumpstead R, O’Brien M, Lee A, Meltzer H. Psychiatric morbidity among adults living in private households, 2000. London: The Stationery Office; 2001.

52. Subramaniam M, Abdin E, Vaingankar JA, Shafie S, Chua HC, Tan WM, et al. Minding the treatment gap: results of the Singapore Mental Health Study. Soc Psychiatry Psychiatr Epidemiol. 2020;55(11):1415-24.

53. World Health Organization. Health in 2015: from MDGs, Millennium Development Goals to SDGs, Sustainable Development Goals. Geneva: World Health Organization; 2015.

54. Duhoux A, Fournier L, Nguyen CT, Roberge P, Beveridge R. Guideline concordance of treatment for depressive disorders in Canada. Soc Psychiatry Psychiatr Epidemiol. 2009;44(5):385-92.

55. Singleton N, Bumpstead R, O'Brien M, Lee A, Meltzer H. Psychiatric morbidity among adults living in private households, 2000. Int Rev Psychiatry. 2003;15(1-2):65-73.

56. Kessler RC, Berglund P, Demler O, Jin R, Koretz D, Merikangas KR, et al. The epidemiology of major depressive disorder: results from the National Comorbidity Survey Replication (NCS-R). JAMA. 2003;289(23):3095-105.

57. Goldney R, Fisher L, Dal Grande E, Taylor A, Hawthorne G. Have education and publicity about depression made a difference? Comparison of prevalence, service use and excess costs in South Australia: 1998 and 2004. Australian and New Zealand Journal of Psychiatry. 2007;41(1):38-53.

58. Andreas S, Dehoust M, Volkert J, Schulz H, Sehner S, Suling A, et al. Affective disorders in the elderly in different European countries: Results from the MentDis_ICF65+ study. PLoS One. 2019;14(11):e0224871.

59. Briggs R, Tobin K, Kenny RA, Kennelly SP. What is the prevalence of untreated depression and death ideation in older people? Data from the Irish Longitudinal Study on Aging. International psychogeriatrics. 2018;30(9):1393-401.

60. Charlson F, Diminic S, Choulamany C, Santomauro D, Raja S, Whiteford H. The prevalence and service utilisation associated with mental and substance use disorders in Lao Peoples Democratic Republic: findings from a cross-sectional survey. Epidemiology and psychiatric sciences. 2019;28(1):54.

61. Cia AH, Stagnaro JC, Aguilar-Gaxiola S, Sustas S, Serfaty E, Nemirovsky M, et al. Twelve-month utilization rates and adequacy of treatment for mental health and substance use disorders in Argentina. Brazilian Journal of Psychiatry. 2018;41:238-44.

62. Eustache E, Gerbasi ME, Smith Fawzi MC, Fils-Aimé JR, Severe J, Raviola GJ, et al. High burden of mental illness and low utilization of care among school-going youth in Central Haiti: A window into the youth mental health treatment gap in a low-income country. International Journal of Social Psychiatry. 2017;63(3):261-74.

63. Fleury MJ, Grenier G, Bamvita JM, Perreault M, Caron J. Variables associated with perceived unmet need for mental health care in a Canadian epidemiologic catchment area. Psychiatr Serv. 2016;67(1):78-85.

64. Hengartner MP, Angst F, Ajdacic-Gross V, Rössler W, Angst J. Treated versus non-treated subjects with depression from a 30-year cohort study: prevalence and clinical covariates. Eur Arch Psychiatry Clin Neurosci. 2016;266(2):173-80.

65. Johnson SE, Lawrence D, Hafekost J, Saw S, Buckingham WJ, Sawyer M, et al. Service use by Australian children for emotional and behavioural problems: Findings from the second Australian Child and Adolescent Survey of Mental Health and Wellbeing. Australian & New Zealand Journal of Psychiatry. 2016;50(9):887-98.

66. Jörg F, Visser E, Ormel J, Reijneveld SA, Hartman CA, Oldehinkel AJ. Mental health care use in adolescents with and without mental disorders. European Child & Adolescent Psychiatry. 2016;25(5):501-8.

67. Lu W. Adolescent depression: national trends, risk factors, and healthcare disparities. American journal of health behavior. 2019;43(1):181-94.

68. Maske UE, Buttery AK, Beesdo-Baum K, Riedel-Heller S, Hapke U, Busch MA. Prevalence and correlates of DSM-IV-TR major depressive disorder, self-reported diagnosed depression and current depressive symptoms among adults in Germany. Journal of affective disorders. 2016;190:167-77.

69. Sawyer MG, Reece CE, Sawyer AC, Hiscock H, Lawrence D. Adequacy of treatment for child and adolescent mental disorders in Australia: A national study. Aust N Z J Psychiatry. 2019;53(4):326-35.

70. Nübel J, Müllender S, Hapke U, Jacobi F. Epidemie der Depression?: Prävalenzentwicklung und Inanspruchnahme von Hilfs-und Versorgungsangeboten. Der Nervenarzt. 2019;90(11).

71. Olfson M, Blanco C, Wall MM, Liu SM, Grant BF. Treatment of Common Mental Disorders in the United States: Results From the National Epidemiologic Survey on Alcohol and Related Conditions-III. J Clin Psychiatry. 2019;80(3).

72. ten Have M, de Graaf R, Van Dorsselaer S, Tuithof M, Kleinjan M, Penninx BW. Recurrence and chronicity of major depressive disorder and their risk indicators in a population cohort. Acta Psychiatrica Scandinavica. 2018;137(6):503-15.

73. Vrublevska J, Trapencieris M, Snikere S, Grinberga D, Velika B, Pudule I, et al. The 12-month prevalence of depression and health care utilization in the general population of Latvia. J Affect Disord. 2017;210:204-10.

74. Johnson SE, Lawrence D, Sawyer M, Zubrick SR. Mental disorders in Australian 4- to 17- year olds: Parent-reported need for help. Aust N Z J Psychiatry. 2018;52(2):149-62.

75. Department of Health and Ageing (Australia), World Health Organization (WHO). Australia World Health Survey 2003. Geneva, Switzerland: World Health Organization (WHO), 2005.

76. World Health Organization (WHO). Austria World Health Survey 2003. Geneva, Switzerland: World Health Organization (WHO), 2005.

77. World Health Organization (WHO). Bangladesh World Health Survey 2003. Geneva, Switzerland: World Health Organization (WHO), 2005.

78. World Health Organization (WHO). Belgium World Health Survey 2002. Geneva, Switzerland: World Health Organization (WHO), 2005.

79. World Health Organization (WHO). Bosnia and Herzegovina World Health Survey 2003. Geneva, Switzerland: World Health Organization (WHO), 2005.

80. Center for Scientific and Technological Information, Oswaldo Cruz Foundation and World Health Organization (WHO). Brazil World Health Survey 2003. Geneva, Switzerland: World Health Organization (WHO), 2005.

81. World Health Organization (WHO). Burkina Faso World Health Survey 2002-2003. Geneva, Switzerland: World Health Organization (WHO), 2005.

82. World Health Organization (WHO). Chad World Health Survey 2003. Geneva, Switzerland: World Health Organization (WHO), 2005.

83. World Health Organization (WHO). China World Health Survey 2002. Geneva, Switzerland: World Health Organization (WHO), 2005.

84. World Health Organization (WHO). Comoros World Health Survey 2003. Geneva, Switzerland: World Health Organization (WHO), 2005.

85. World Health Organization (WHO). Côte d'Ivoire World Health Survey 2003. Geneva, Switzerland: World Health Organization (WHO), 2005.

86. World Health Organization (WHO). Croatia World Health Survey 2003. Geneva, Switzerland: World Health Organization (WHO), 2005.

87. World Health Organization (WHO). Czech Republic World Health Survey 2002-2003. Geneva, Switzerland: World Health Organization (WHO), 2005.

88. World Health Organization (WHO). Denmark World Health Survey 2003. Geneva, Switzerland: World Health Organization (WHO), 2005.

89. World Health Organization (WHO). Dominican Republic World Health Survey 2003. Geneva, Switzerland: World Health Organization (WHO), 2005.

90. World Health Organization (WHO). Ecuador World Health Survey 2003. Geneva, Switzerland: World Health Organization (WHO), 2005.

91. World Health Organization (WHO). Estonia World Health Survey 2003. Geneva, Switzerland: World Health Organization (WHO), 2005.

92. World Health Organization (WHO). France World Health Survey 2003. Geneva, Switzerland: World Health Organization (WHO), 2005.

93. World Health Organization (WHO). Georgia World Health Survey 2003. Geneva, Switzerland: World Health Organization (WHO), 2005.

94. World Health Organization (WHO). Germany World Health Survey 2003. Geneva, Switzerland: World Health Organization (WHO), 2005.

95. World Health Organization (WHO). Ghana World Health Survey 2003. Geneva, Switzerland: World Health Organization (WHO), 2005.

96. World Health Organization (WHO). Greece World Health Survey 2003. Geneva, Switzerland: World Health Organization (WHO), 2005.

97. International Institute for Population Sciences (India), World Health Organization (WHO). India World Health Survey 2003. Geneva, Switzerland: World Health Organization (WHO), 2005.

98. World Health Organization (WHO). Ireland World Health Survey 2003. Geneva, Switzerland: World Health Organization (WHO), 2005.

99. World Health Organization (WHO). Italy World Health Survey 2003. Geneva, Switzerland: World Health Organization (WHO), 2005.

100. World Health Organization (WHO). Kazakhstan World Health Survey 2002-2003. Geneva, Switzerland: World Health Organization (WHO), 2005.

101. World Health Organization (WHO). Kenya World Health Survey 2004. Geneva, Switzerland: World Health Organization (WHO), 2005.

102. World Health Organization (WHO). Laos World Health Survey 2003.

103. World Health Organization (WHO). Latvia World Health Survey 2003. Geneva, Switzerland: World Health Organization (WHO), 2005.

104. World Health Organization (WHO). Luxembourg World Health Survey 2003. Geneva, Switzerland: World Health Organization (WHO), 2005.

105. World Health Organization (WHO). Malawi World Health Survey 2003. Geneva, Switzerland: World Health Organization (WHO), 2005.

106. World Health Organization (WHO). Malaysia World Health Survey 2003. Geneva, Switzerland: World Health Organization (WHO), 2005.

107. World Health Organization (WHO). Mali World Health Survey 2003. Geneva, Switzerland: World Health Organization (WHO), 2005.

108. World Health Organization (WHO). Mauritania World Health Survey 2003. Geneva, Switzerland: World Health Organization (WHO), 2005.

109. World Health Organization (WHO). Mauritius World Health Survey 2003. Geneva, Switzerland: World Health Organization (WHO), 2005.

110. World Health Organization (WHO). Morocco World Health Survey 2003. Geneva, Switzerland: World Health Organization (WHO), 2005.

111. World Health Organization (WHO). Myanmar World Health Survey 2003. Geneva, Switzerland: World Health Organization (WHO), 2005.

112. World Health Organization (WHO). Namibia World Health Survey 2003. Geneva, Switzerland: World Health Organization (WHO), 2005.

113. World Health Organization (WHO). Netherlands World Health Survey 2004. Geneva, Switzerland: World Health Organization (WHO), 2005.

114. World Health Organization (WHO). Norway World Health Survey 2003. Geneva, Switzerland: World Health Organization (WHO), 2005.

115. World Health Organization (WHO). Pakistan World Health Survey 2003-2004. Geneva, Switzerland: World Health Organization (WHO), 2005.

116. World Health Organization (WHO). Paraguay World Health Survey 2002-2003. Geneva, Switzerland: World Health Organization (WHO), 2005.

117. World Health Organization (WHO). Philippines World Health Survey 2003. Geneva, Switzerland: World Health Organization (WHO), 2005.

118. World Health Organization (WHO). Portugal World Health Survey 2003. Geneva, Switzerland: World Health Organization (WHO), 2006.

119. World Health Organization (WHO). Congo World Health Survey 2003. Geneva, Switzerland: World Health Organization (WHO), 2005.

120. World Health Organization (WHO). Russia World Health Survey 2003. Geneva, Switzerland: World Health Organization (WHO), 2005.

121. World Health Organization (WHO). Senegal World Health Survey 2003. Geneva, Switzerland: World Health Organization (WHO), 2005.

122. World Health Organization (WHO). Slovakia World Health Survey 2003. Geneva, Switzerland: World Health Organization (WHO), 2005.

123. World Health Organization (WHO). Slovenia World Health Survey 2003. Geneva, Switzerland: World Health Organization (WHO), 2005.

124. World Health Organization (WHO). South Africa World Health Survey 2002-2003. Geneva, Switzerland: World Health Organization (WHO), 2005.

125. World Health Organization (WHO). Spain World Health Survey 2002-2003. Geneva, Switzerland: World Health Organization (WHO), 2005.

126. World Health Organization (WHO). Sri Lanka World Health Survey 2003. Geneva, Switzerland: World Health Organization (WHO), 2005.

127. World Health Organization (WHO). Swaziland World Health Survey 2003. Geneva, Switzerland: World Health Organization (WHO), 2005.

128. World Health Organization (WHO). Sweden World Health Survey 2003. Geneva, Switzerland: World Health Organization (WHO), 2005.

129. World Health Organization (WHO). Tunisia World Health Survey 2003. Geneva, Switzerland: World Health Organization (WHO), 2005.

130. World Health Organization (WHO). Ukraine World Health Survey 2002-2003. Geneva, Switzerland: World Health Organization (WHO), 2005.

131. World Health Organization (WHO). United Arab Emirates World Health Survey 2003. Geneva, Switzerland: World Health Organization (WHO), 2005.

132. World Health Organization (WHO). United Kingdom World Health Survey 2004. Geneva, Switzerland: World Health Organization (WHO), 2005.

133. World Health Organization (WHO). Uruguay World Health Survey 2002-2003. Geneva, Switzerland: World Health Organization (WHO), 2005.

134. World Health Organization (WHO). Vietnam World Health Survey 2002-2003. Geneva, Switzerland: World Health Organization (WHO), 2005.

135. World Health Organization (WHO). Zambia World Health Survey 2003. Geneva, Switzerland: World Health Organization (WHO), 2005.

136. World Health Organization (WHO). Zimbabwe World Health Survey 2003. Geneva, Switzerland: World Health Organization (WHO), 2005.
