# Supplementary material for: The global gap in treatment coverage for major depressive disorder in 84 countries from 2000–2019: A systematic review and Bayesian meta-regression analysis
Source: PLoS Med. 2022 Feb 15;19(2):e1003901. doi: 10.1371/journal.pmed.1003901 (PMC8846511; doi:10.1371/journal.pmed.1003901)
Supplement: S1 Checklist — (DOCX) [file pmed.1003901.s002.docx]

| **Section and Topic** | **Item #** | **Checklist item** | **Location where item is reported** |
| --- | --- | --- | --- |
| **TITLE** |  | | |
| Title | 1 | Identify the report as a systematic review. | Title |
| **ABSTRACT** |  | | |
| Abstract | 2 | See the PRISMA 2020 for Abstracts checklist. | Manuscript Abstract section (all paragraphs) |
| **INTRODUCTION** |  | | |
| Rationale | 3 | Describe the rationale for the review in the context of existing knowledge. | Manuscript Introduction section (all paragraphs) |
| Objectives | 4 | Provide an explicit statement of the objective(s) or question(s) the review addresses. | Manuscript Introduction section (fifth paragraph) |
| **METHODS** |  | | |
| Eligibility criteria | 5 | Specify the inclusion and exclusion criteria for the review and how studies were grouped for the syntheses. | Manuscript Methods section, Data sources and search strategy subsection (third paragraph) |
| Information sources | 6 | Specify all databases, registers, websites, organisations, reference lists and other sources searched or consulted to identify studies. Specify the date when each source was last searched or consulted. | Manuscript Methods section, Data sources and search strategy subsection (second paragraph) |
| Search strategy | 7 | Present the full search strategies for all databases, registers and websites, including any filters and limits used. | Appendix Section 1 |
| Selection process | 8 | Specify the methods used to decide whether a study met the inclusion criteria of the review, including how many reviewers screened each record and each report retrieved, whether they worked independently, and if applicable, details of automation tools used in the process. | Manuscript Methods section, Data sources and search strategy subsection (second paragraph) |
| Data collection process | 9 | Specify the methods used to collect data from reports, including how many reviewers collected data from each report, whether they worked independently, any processes for obtaining or confirming data from study investigators, and if applicable, details of automation tools used in the process. | Manuscript Methods section, Data sources and search strategy subsection (second paragraph), Data collection and processing subsection (first paragraph) |
| Data items | 10a | List and define all outcomes for which data were sought. Specify whether all results that were compatible with each outcome domain in each study were sought (e.g. for all measures, time points, analyses), and if not, the methods used to decide which results to collect. | Manuscript Methods section, Data sources and search strategy subsection (third paragraph), Data collection and processing subsection (first paragraph) |
|  | 10b | List and define all other variables for which data were sought (e.g. participant and intervention characteristics, funding sources). Describe any assumptions made about any missing or unclear information. | Manuscript Methods section Tables 1 and 2. Data collection and processing subsection (first paragraph) |
| Study risk of bias assessment | 11 | Specify the methods used to assess risk of bias in the included studies, including details of the tool(s) used, how many reviewers assessed each study and whether they worked independently, and if applicable, details of automation tools used in the process. | Manuscript Methods section, Statistical Analysis subsection (first paragraph), Manuscript Methods section, Data sources and search strategy (second paragraph) |
| Effect measures | 12 | Specify for each outcome the effect measure(s) (e.g. risk ratio, mean difference) used in the synthesis or presentation of results. | Manuscript Methods section, Data sources and search strategy subsection (third paragraph) |
| Synthesis methods | 13a | Describe the processes used to decide which studies were eligible for each synthesis (e.g. tabulating the study intervention characteristics and comparing against the planned groups for each synthesis (item #5)). | Manuscript Methods section. Data sources and search strategy subsection (third paragraph) |
|  | 13b | Describe any methods required to prepare the data for presentation or synthesis, such as handling of missing summary statistics, or data conversions. | Manuscript Methods section, Data collection and processing subsection (first paragraph), Statistical Analyses subsection (all paragraphs) |
|  | 13c | Describe any methods used to tabulate or visually display results of individual studies and syntheses. | Manuscript Methods section, Statistical Analyses subsection (last line) |
|  | 13d | Describe any methods used to synthesize results and provide a rationale for the choice(s). If meta-analysis was performed, describe the model(s), method(s) to identify the presence and extent of statistical heterogeneity, and software package(s) used. | Manuscript Methods section, Statistical Analyses subsection (all paragraphs) |
|  | 13e | Describe any methods used to explore possible causes of heterogeneity among study results (e.g. subgroup analysis, meta-regression). | Manuscript Methods section, Statistical Analyses subsection (first paragraph) |
|  | 13f | Describe any sensitivity analyses conducted to assess robustness of the synthesized results. | Manuscript Methods section, Statistical Analyses subsection (first paragraph) |
| Reporting bias assessment | 14 | Describe any methods used to assess risk of bias due to missing results in a synthesis (arising from reporting biases). | Manuscript Methods section, Statistical Analyses subsection (first paragraph) |
| Certainty assessment | 15 | Describe any methods used to assess certainty (or confidence) in the body of evidence for an outcome. | Manuscript Methods section, Statistical Analyses subsection (all paragraphs) |
| **RESULTS** |  | | |
| Study selection | 16a | Describe the results of the search and selection process, from the number of records identified in the search to the number of studies included in the review, ideally using a flow diagram. | Manuscript Results section first paragraph and Table 3, Appendix Section 2. |
|  | 16b | Cite studies that might appear to meet the inclusion criteria, but which were excluded, and explain why they were excluded. | Appendix Section 2. |
| Study characteristics | 17 | Cite each included study and present its characteristics. | Appendix Section 5. |
| Risk of bias in studies | 18 | Present assessments of risk of bias for each included study. | Manuscript Results section, Regression Results for MDD data subsection, Funnel plots |
| Results of individual studies | 19 | For all outcomes, present, for each study: (a) summary statistics for each group (where appropriate) and (b) an effect estimate and its precision (e.g. confidence/credible interval), ideally using structured tables or plots. | Appendix Section 5. |
| Results of syntheses | 20a | For each synthesis, briefly summarise the characteristics and risk of bias among contributing studies. | Manuscript Results section, Regression Results for MDD data subsection (all paragraphs) |
|  | 20b | Present results of all statistical syntheses conducted. If meta-analysis was done, present for each the summary estimate and its precision (e.g. confidence/credible interval) and measures of statistical heterogeneity. If comparing groups, describe the direction of the effect. | Manuscript Results section, Regression Results for MDD data subsection (all paragraphs) |
|  | 20c | Present results of all investigations of possible causes of heterogeneity among study results. | Manuscript Results section, Regression Results for MDD data subsection (all paragraphs) |
|  | 20d | Present results of all sensitivity analyses conducted to assess the robustness of the synthesized results. | Appendix Section 4. |
| Reporting biases | 21 | Present assessments of risk of bias due to missing results (arising from reporting biases) for each synthesis assessed. | Manuscript Results section, Regression Results for MDD data subsection, Funnel plots |
| Certainty of evidence | 22 | Present assessments of certainty (or confidence) in the body of evidence for each outcome assessed. | Manuscript Results section, Regression Results for MDD data subsection (all paragraphs and Table 4) |
| **DISCUSSION** |  | | |
| Discussion | 23a | Provide a general interpretation of the results in the context of other evidence. | Manuscript Discussion section paragraphs 1-4 |
|  | 23b | Discuss any limitations of the evidence included in the review. | Manuscript Discussion section paragraphs 7-8 |
|  | 23c | Discuss any limitations of the review processes used. | Manuscript Discussion section paragraphs 7-8 |
|  | 23d | Discuss implications of the results for practice, policy, and future research. | Manuscript Discussion section paragraphs 3-6, 8, Conclusion section. |
| **OTHER INFORMATION** |  | | |
| Registration and protocol | 24a | Provide registration information for the review, including register name and registration number, or state that the review was not registered. | Manuscript Methods section, Data sources and search strategy subsection, paragraph 2. |
|  | 24b | Indicate where the review protocol can be accessed, or state that a protocol was not prepared. | Manuscript Methods section, Data sources and search strategy subsection, paragraph 2. |
|  | 24c | Describe and explain any amendments to information provided at registration or in the protocol. | NA |
| Support | 25 | Describe sources of financial or non-financial support for the review, and the role of the funders or sponsors in the review. | Manuscript Funding subsection |
| Competing interests | 26 | Declare any competing interests of review authors. | Manuscript Competing Interests subsection |
| Availability of data, code and other materials | 27 | Report which of the following are publicly available and where they can be found: template data collection forms; data extracted from included studies; data used for all analyses; analytic code; any other materials used in the review. | Manuscript Data Availability subsection |
